# Megakaryocytes transfer mitochondria to bone marrow mesenchymal stromal cells to lower platelet activation

**DOI:** 10.1172/JCI189801

**Published:** 2025-02-27

**Authors:** Chengjie Gao, Yitian Dai, Paul A. Spezza, Paul Boasiako, Alice Tang, Giselle Rasquinha, Hui Zhong, Bojing Shao, Yunfeng Liu, Patricia A. Shi, Cheryl A. Lobo, Xiuli An, Anqi Guo, William B. Mitchell, Deepa Manwani, Karina Yazdanbakhsh, Avital Mendelson

**Affiliations:** 1 Laboratory of Membrane Biology,; 2 Laboratory of Stem Cell Biology and Engineering Research,; 3 Laboratory of Immune Regulation,; 4 Laboratory of Vascular Inflammation and Thrombosis Research,; 5 Laboratory of Complement Biology,; 6 Sickle Cell Clinical Research Program, and; 7 Laboratory of Blood Borne Parasites, New York Blood Center, New York, New York, USA.; 8 Department of Pediatrics, Montefiore Health Center, Albert Einstein College of Medicine, Children’s Hospital at Montefiore, Bronx, New York, USA.

**Keywords:** Hematology, Stem cells, Bone marrow, Mouse stem cells, Platelets

## Abstract

Newly produced platelets acquire a low activation state, but whether the megakaryocyte plays a role in this outcome has not been fully uncovered. Mesenchymal stem cells (MSCs) were previously shown to promote platelet production and lower platelet activation. We found that healthy megakaryocytes transfer mitochondria to MSCs, which is mediated by connexin 43 (Cx43) gap junctions on MSCs and leads to platelets at a low energetic state with increased LYN activation, characteristic of resting platelets with increased LYN activation, characteristic of resting platelets. On the contrary, MSCs have a limited ability to transfer mitochondria to megakaryocytes. Sickle cell disease (SCD) is characterized by hemolytic anemia and results in heightened platelet activation, contributing to numerous disease complications. Platelets in SCD mice and human samples had a heightened energetic state with increased glycolysis. MSC exposure to heme in SCD led to decreased Cx43 expression and a reduced ability to uptake mitochondria from megakaryocytes. This prevented LYN activation in platelets and contributed to increased platelet activation at steady state. Altogether, our findings demonstrate an effect of hemolysis in the microenvironment leading to increased platelet activation in SCD. These findings have the potential to inspire new therapeutic targets to relieve thrombosis-related complications of SCD and other hemolytic conditions.

## Abstract 2

Megakaryocytes transfer mitochondria to MSCs leading to lower glycolysis and decreased platelet activation. In sickle cell disease, mitochondria transfer is reduced promoting heightened platelet activation.

## Graphical abstract

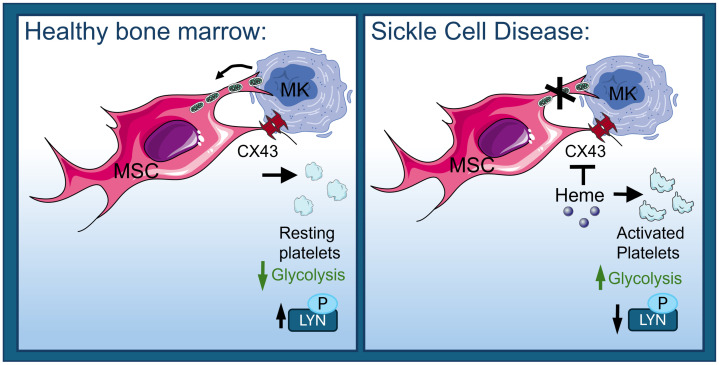

## Introduction

The bone marrow niche for regulating megakaryocyte (MK) function and preventing platelet activation remains poorly understood. We recently identified a role for mesenchymal stem cells (MSCs) from both bone marrow and umbilical cord tissue, which can lower baseline platelet activation (1), though the mechanism remains unclear. Platelets in a skin wound healing model can transfer mitochondria to MSCs leading to a proangiogenic phenotype and enhanced tissue repair (2). While intercellular mitochondria transfer has been demonstrated between MSCs and various cell types, such as hematopoietic stem and progenitor cells, immune cells, or alveolar epithelia (3–7), whether mature MKs can also transfer mitochondria to MSCs, leading to the production of platelets with low baseline activation, has not been explored.

Sickle cell disease (SCD) is an inherited blood disorder, which results in the polymerization of hemoglobin S and sickling of red blood cells, leading to hemolysis along with thrombotic and inflammatory alterations (8–11). Heightened platelet activation in SCD is believed to contribute to numerous complications, including stroke, thromboembolism, and cardiovascular complications, among others (8, 12, 13). Platelet inhibition with aspirin treatment was investigated in preventing vaso-occlusion in SCD, but clinical trial results were mainly negative (14–16). A recent study identified reduced platelet aggregation following treatment with crizanlizumab (17), which targets P selectin on the surface of platelets and endothelial cells to prevent pain crises, though further investigation is ongoing to confirm its therapeutic benefits. A trial of P2Y_12_ inhibition with prasugrel also did not identify sufficient benefits for reducing vaso-occlusive crisis events (18). While primary clinical trials of ticagrelor P2Y_12_ inhibitor displayed promising results, phase III studies were halted due to lack of therapeutic benefit (19, 20). Thus, alternative antiplatelet and antithrombotic strategies for treating SCD are needed. Previously we found that SCD MSCs have severely impaired functionality, including decreased expression of hematopoietic maintenance genes and impaired self-renewal capacity (21). As a consequence, the ability of SCD MSCs to maintain hematopoietic stem cells in the bone marrow was also lowered (21). Whether SCD MSCs also have a reduced ability to communicate with MKs leading to increased platelet activation remains unknown.

Here, we examined the mechanism by which bone marrow–derived MSCs can promote low baseline platelet activation at steady state. We assessed mitochondrial transfer between MKs and MSCs, mediated by connexin 43 (Cx43) gap junctions, leading to metabolic changes among MKs and MSCs. This in turn translated to activation of LYN signaling and low baseline activation levels in platelets. MKs in SCD were also examined for their metabolic changes due to dysfunctional MSCs leading to increased platelet activation. Reducing baseline platelet activation in SCD could have cascading effects, resulting in lower inflammation and reduced complications of the disease.

## Results

### MKs transfer mitochondria to MSCs.

**Figure 1:**
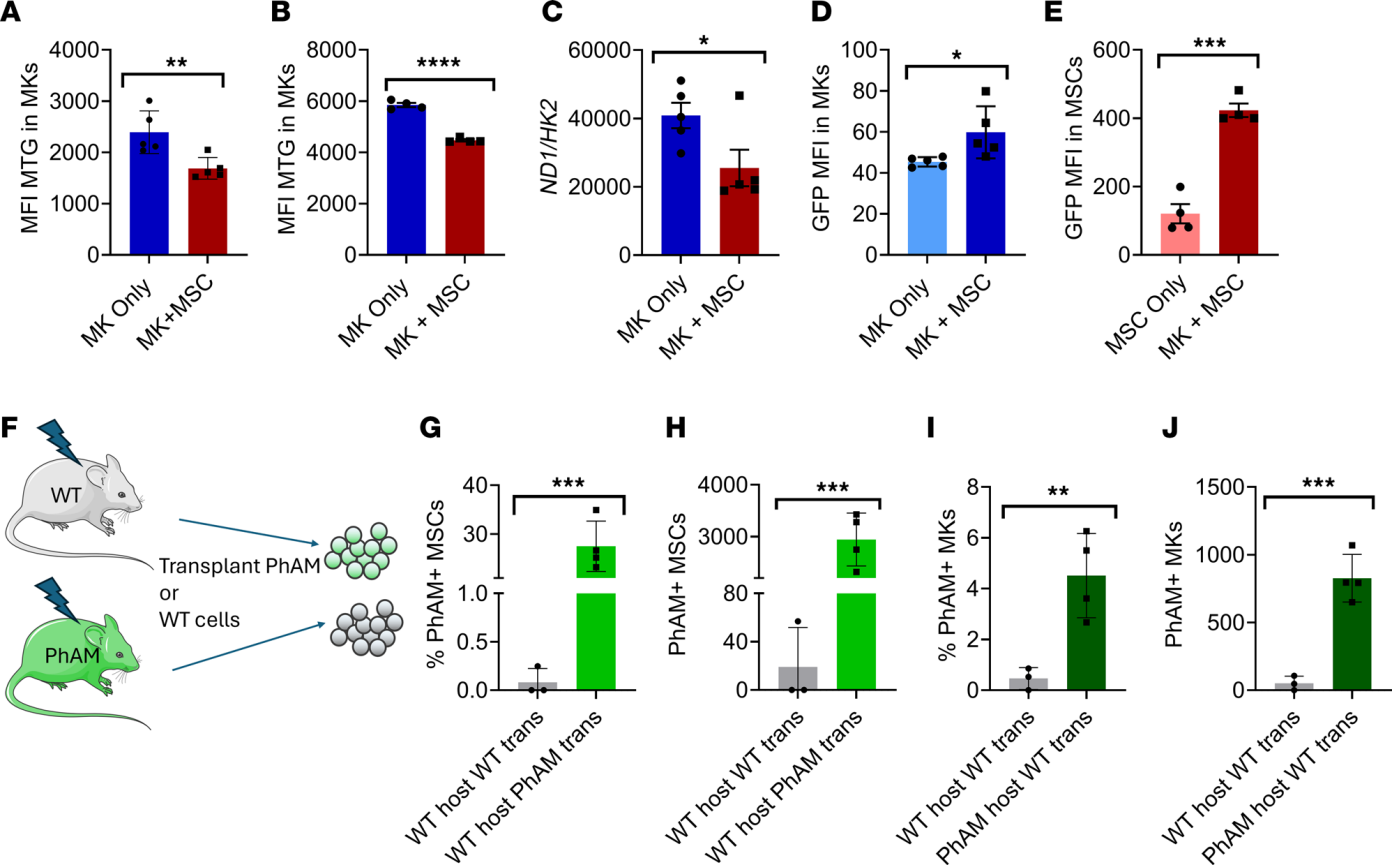
Megakaryocytes transfer mitochondria to mesenchymal stem cells. C57BL/6 murine MKs were cultured together with C57BL/6 MSCs or cultured alone. By flow cytometry, MKs from cocultures were analyzed for (**A**) mean fluorescence intensity (MFI) of mitotracker green (MTG) (*n* = 5) and (**B**) MFI of MTG from human cord blood CD34^+^ cell-derived MKs cocultured with human bone marrow MSCs (*n* = 4). (**C**) RT-PCR expression of Nd1/Hk2 in murine MKs from cocultures (*n* = 5). (**D**) C57BL/6 murine MKs were cultured together with MSCs from *PhAM-floxed;E2a-cre* mice or cultured alone. MFI of mitochondria PhAM signal in MKs by flow cytometry (*n* = 5). (**E**) Wild-type murine MSCs were cultured together with murine MKs from *PhAM-floxed;E2a-cre* mice or cultured alone. MFI of PhAM mitochondrial signal in MSCs by flow cytometry (*n* = 4). (**F**) Schematic of transplant design in which C57BL/6 mice were transplanted with *PhAM-floxed;E2a-cre* bone marrow cells or *PhAM-floxed;E2a-cre* mice were transplanted with C57BL/6 cells. (**G**) Bone marrow MSCs analyzed from transplanted mice for percentage PhAM^+^ MSCs and (**H**) numbers of PhAM^+^ MSCs per femur. (**I**) Bone marrow MKs analyzed from transplanted mice for percentage PhAM^+^ MKs and (**J)** numbers of PhAM^+^ MKs per femur. *n* = 3–4 mice for **G**–**J**. *P ≤ 0.05, **P ≤ 0.01, ***P ≤ 0.001, ****P ≤ 0.0001. Data were analyzed with 2-tailed, unpaired Student’s *t* test. Data are presented as mean ± SEM.

Given that platelet mitochondria serve functions in platelet activation, metabolism, and ATP production (22), we were interested to investigate whether changes to the mitochondria may occur at the MK level due to MSC-MK interactions. Primary murine wild-type MKs cultured together with wild-type murine MSCs were found to have reduced mitochondria content by mitotracker green analysis (Figure 1A). Human cord blood CD34^+^ cell–derived MKs cultured together with human bone marrow MSCs also showed reduced mitochondrial content following coculture (Figure 1B). MKs from murine MSC-MK cocultures were further found to have a lower *Nd1/Hk2* ratio by RT-PCR (Figure 1C) compared with MKs cultured alone, further confirming a reduced mitochondrial content. We examined the possibility of mitochondrial transfer occurring between MKs and MSCs using cells from a *PhAM-floxed;E2a-cre* mouse model in which mitochondria are fluorescently labeled with GFP (23). Coculture of wild-type MKs with *PhAM-floxed* MSCs was conducted to examine mitochondrial transfer from MSCs to MKs. This resulted in only a slight increase in GFP signal within cocultured MKs (Figure 1D). In a second coculture, we assessed whether there may be mitochondrial transfer occurring from MKs to MSCs using MKs from *PhAM-floxed;E2a-cre* mice with MSCs from wild-type mice. Surprisingly, we found a significant increase in GFP signal among wild-type MSCs from the coculture (Figure 1E and Supplemental Videos 1 and 2). Cultures were analyzed at 18 hours because a time-course study revealed mitochondrial transfer levels from MKs at this time point were among maximal levels (Supplemental Figure 1A). To further verify mitochondrial transfer, murine wild-type MKs were prelabeled with PKmito deep red mitochondria dye followed by coculture with wild-type MSCs. MKs and platelets from cocultures displayed a reduction in mitochondrial signal compared with MKs cultured alone (Supplemental Figure 1, B and C), while MSCs acquired mitochondrial signal from cocultured MKs (Supplemental Figure 1D). Importantly, isolated MKs and MSCs from cocultures remained pure and devoid of cross-contamination (Supplemental Figure 1, E–G). When MKs were pretreated with Oligomycin A to inhibit mitochondrial ATP synthetase, mitochondrial transfer to MSCs was abrogated (Supplemental Figure 1H). Overall, this suggests that MKs are transferring mitochondria to MSCs while MKs receive a minimal amount of mitochondria from MSCs. To examine mitochondrial uptake in vivo, we transplanted wild-type mice with bone marrow from *PhAM-floxed;E2a-cre* mice and transplanted *PhAM-floxed;E2a-cre* mice with bone marrow from wild-type mice (Figure 1F). Bone marrow MSC and MK GFP intensity in *PhAM-floxed* mice were comparable at baseline (Supplemental Figure 2A), and PhAM GFP^+^ signal could be clearly distinguished in comparison with wild-type mice (Supplemental Figure 2B). At 12 weeks after transplant, approximately 30% of wild-type host MSCs were found to contain GFP signal from transplanted *PhAM-floxed* bone marrow (Figure 1, G and H, and Supplemental Figure 2C), whereas only 4% of MKs from transplanted wild-type bone marrow contained host mitochondrial GFP signal (Figure 1, I and J, and Supplemental Figure 2D), further suggesting a biased directionality of mitochondrial transfer.

### MKs undergo metabolic changes after transferring mitochondria.

**Figure 2:**
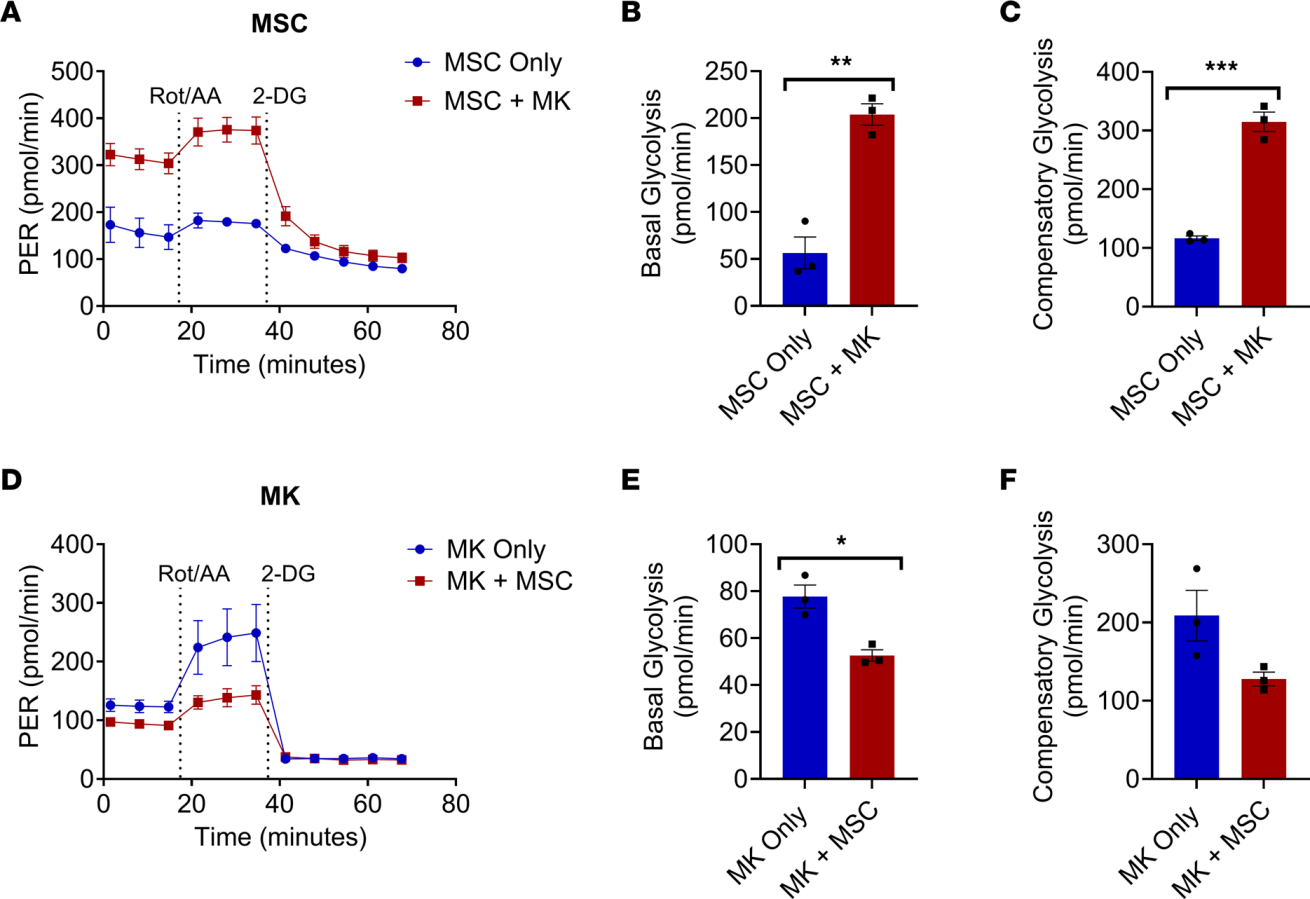
Metabolic changes to megakaryocytes and MSCs following coculture. C57BL/6 murine MKs were cultured together with C57BL/6 MSCs or cultured alone. (**A**) A representative Seahorse glycolytic rate assay conducted on MSCs following coculture. (**B**) Basal glycolysis levels of MSCs from coculture. (**C**) Compensatory glycolysis of MSCs following coculture. (**D**) Representative Seahorse glycolytic rate assay conducted on MKs following coculture. (**E**) Basal glycolysis levels of MKs from coculture. (**F**) Compensatory glycolysis of MKs following coculture. *n* = 3. *P ≤ 0.05, **P ≤ 0.01, ***P ≤ 0.001. Data were analyzed with 2-tailed, unpaired Student’s *t* test. Data are presented as mean ± SEM.

To examine if MKs cocultured with MSCs led to alterations in the metabolic properties of both cell types, we conducted a Seahorse Glycolytic Rate Assay (Agilent). Following coculture with MKs, MSCs were found to be more glycolytic compared with MSCs cultured alone (Figure 2A), including significantly increased basal glycolysis (Figure 2B) and compensatory glycolysis (Figure 2C). Interestingly, MKs cocultured with MSCs had a reduction in glycolysis compared with MKs cultured alone (Figure 2D), leading to both lower basal glycolysis (Figure 2E) and compensatory glycolysis (Figure 2F). MKs had a reduced ability to uptake glucose following MSC coculture compared with MKs cultured alone (Supplemental Figure 1I) and MKs pretreated with oligomycin following by coculture with MSCs (Supplemental Figure 1I). Given that MKs enhance their mitochondrial biogenesis with increased maturation (24) and the presence of MSCs leads to greater platelet formation by MKs (1), we found an increased MK ratio of ATP/ADP following MSC-MK coculture compared with MKs cultured alone (Supplemental Figure 1J). A high ATP/ADP ratio in cocultured MKs led to inhibition of glycolysis, whereas a decreased ATP/ADP ratio in MKs cultured alone and in MKs pretreated with oligomycin followed by coculture led to a glycolytic phenotype (Supplemental Figure 1, I and J). Thus, MSCs following MK coculture have an enhanced energetic state, whereas MKs following MSC coculture have lowered metabolic activity.

### Cx43 gap junctions on MSCs mediate mitochondrial acceptance.

**Figure 3:**
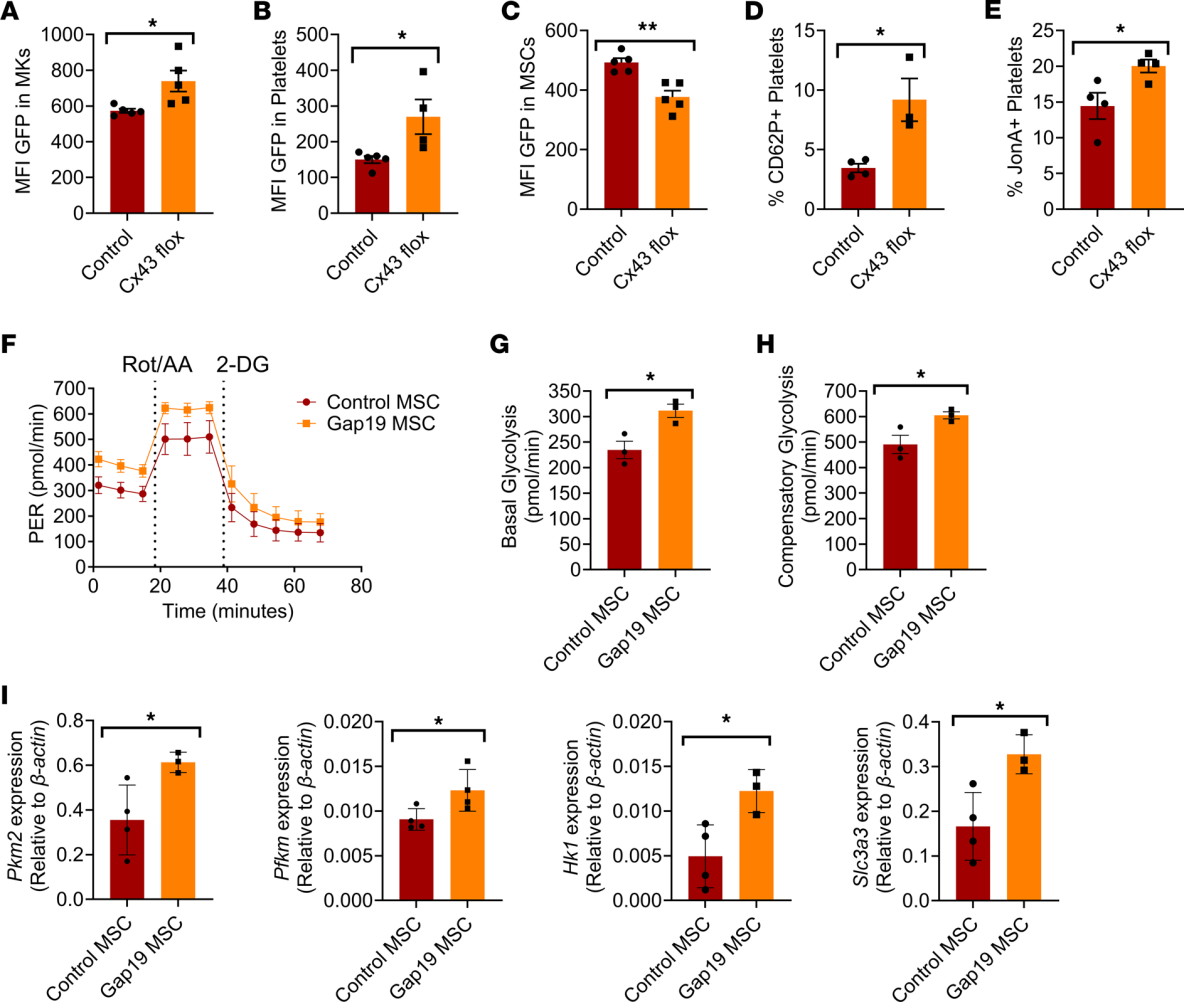
MSC CX43 gap junctions mediate mitochondrial transfer from megakaryocytes. *PhAM-floxed;E2a-cre* murine MKs cultured together with C57BL/6 MSCs or MSCs from *CX43-floxed;Lepr-cre* mice. Following coculture, flow cytometry assessment of (**A**) mean fluorescence intensity (MFI) of PhAM signal in MKs (*n* = 5), (**B**) MFI of PhAM signal in platelets (*n* = 5), (**C**) MFI of PhAM signal in MSCs (*n* = 5), (**D**) CD62P expression on platelets (*n* = 4), and (**E**) JonA (αIIbβ3) expression on platelets (*n* = 4). (**F**) Representative Seahorse glycolytic rate assay conducted on MKs following coculture with wild-type or Gap19-treated MSCs (*n* = 3). (**G**) Basal glycolysis levels of MKs from coculture (*n* = 3). (**H**) Compensatory glycolysis of MKs following coculture (*n* = 3). (**I**) RT-PCR analysis of glycolytic genes (*Pkm2*, *Pfkm*, *Hk1*, and *Slc3a3*) in MKs from cocultures with either C57BL/6 MSCs or C57BL/6 MSCs pretreated with Gap19 to inhibit CX43 expression (*n* = 3–4). *P ≤ 0.05, **P ≤ 0.01. Data were analyzed with 2-tailed, unpaired Student’s *t* test. Data are presented as mean ± SEM.

Cx43 gap junctions have been demonstrated to mediate the transfer of intracellular content between cells (4, 7, 25, 26). Using a mouse model in which Cx43 is selectively depleted from *Lepr^+^* MSCs, we examined whether mitochondrial transfer between MKs and MSCs occurs through Cx43 gap junctions on MSCs. MSCs isolated from conditional Cx43-KO (*Cx43-floxed*) mice were cocultured with MKs from *PhAM-floxed;E2a-cre* mice, which resulted in mitochondrial retention in MKs (Figure 3A) compared with coculture with wild-type MSCs. We also observed increased mitochondrial content in platelets produced within cocultures containing *Cx43-floxed* MSCs compared with cocultures with wild-type MSCs (Figure 3B). *Cx43-floxed* MSCs had less mitochondrial uptake ability compared with wild-type MSCs (Figure 3C). Furthermore, platelets produced in culture with *Cx43-floxed* MSCs had increased expression of platelet activation markers CD62P (Figure 3D) and JonA (Figure 3E) compared with cultures containing wild-type MSCs. Accordingly, we also observed a reduced ability for platelets from *Cx43-floxed* mice to activate and aggregate in response to thrombin (Supplemental Figure 3, A–D) and CRP compared with platelets from wild-type mice (Supplemental Figure 3, E–H). Platelets from Cx43-floxed mice subjected to the T-TAS atheroma chip coagulation assay led to a reduced channel occlusion time compared with control platelets (Supplemental Figure 3I). Platelets from cocultures with Cx43 inhibited MSCs, and healthy MKs were subjected to a flow cytometry–based aggregation assay and displayed a reduced aggregation (27) ability compared with healthy MSC-MK cocultures (Supplemental Figure 3J). Evaluation of the MK glycolytic profile by Seahorse revealed that MKs cocultured with Gap19-treated MSCs to inhibit Cx43 led to increased glycolysis compared with coculture with wild-type MSCs (Figure 3, F–H). Compared with MKs cocultured with control MSCs, coculturing MKs with Gap19-treated MSCs also resulted in increased glucose uptake ability (Supplemental Figure 1I) and reduced intracellular ATP/ADP ratios (Supplemental Figure 1J), which was confirmed by increased expression of glycolysis genes pyruvate kinase M2 (*Pkm2*), phosphofructokinase (*Pkfm*), hexokinase 1 (*Hk1*), and solute carrier family 3 member 1 (*Slc3a3*) by RT-PCR (Figure 3I). Therefore, our data suggest that MSC Cx43 gap junctions may mediate mitochondrial transfer from MKs to MSCs, which in turn leads to downstream alterations in platelet activation.

Given that gap junction–mediated intracellular trafficking requires close proximity (28), we examined the possibility that Cx43 deletion in MSCs leads to altered MK localization away from MSCs compared with wild-type MSC-MK localization. Through whole-mount bone marrow imaging, we found MKs in *Cx43-floxed* mice localized at an average distance of 22.9 μm away from *Lepr^+^* MSCs, whereas MKs in wild-type mice localized at an average distance of 20.8 μm (Supplemental Figure 4) from *Lepr^+^* MSCs. This raises the possibility that increased distance between MKs and MSCs may further prevent mitochondrial transfer between MKs and MSCs due to inhibited Cx43 expression.

### MSCs alter the transcriptome signature of platelets.

**Figure 4:**
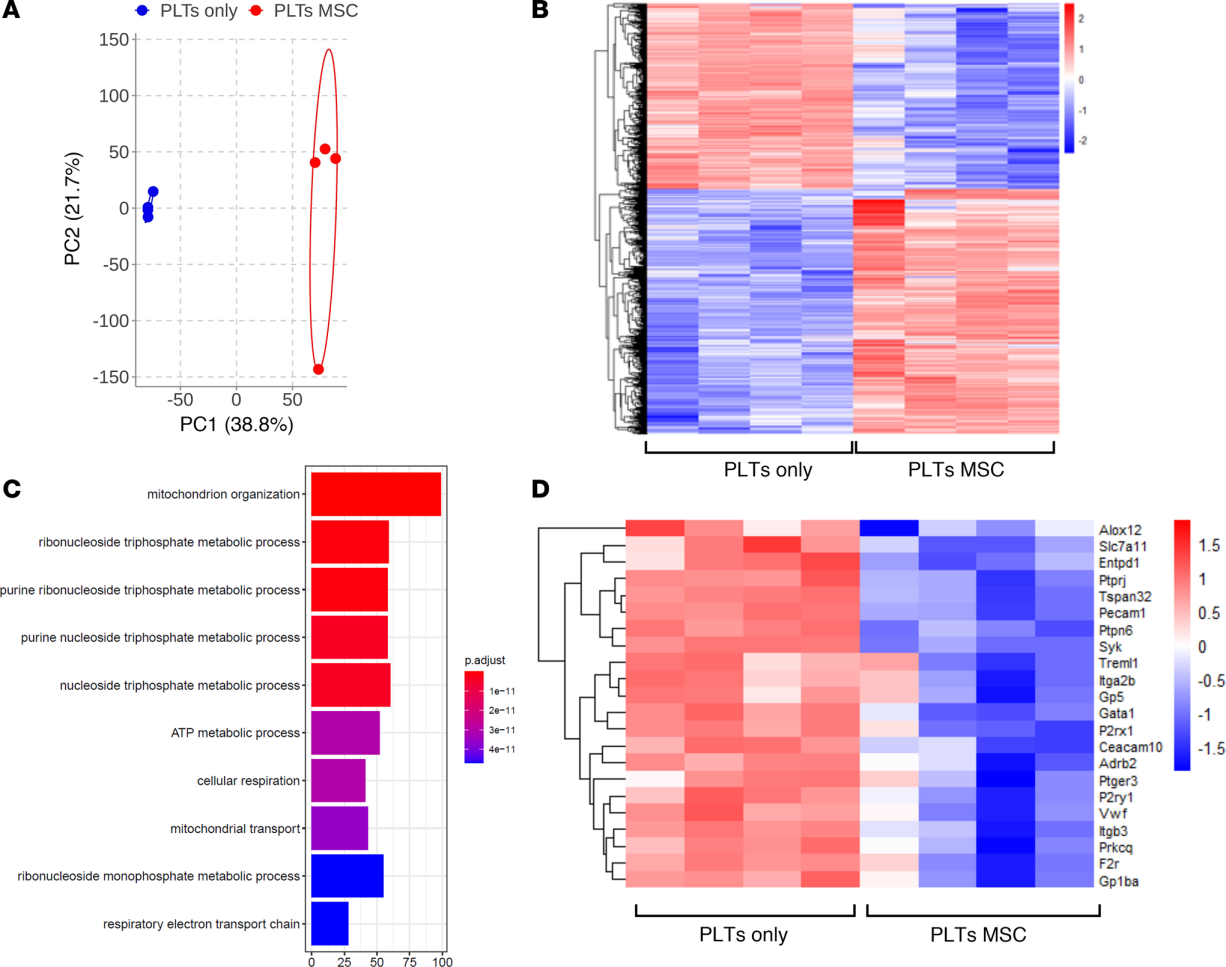
MSCs alter platelet transcriptomics signature. C57BL/6 murine MKs were cultured together with C57BL/6 MSCs or cultured alone. Platelets from cultures were pooled to conduct bulk RNA-Seq analysis (*n* = 4 mice per group). (**A**) Principal component analysis. (**B**) Top differentially expressed genes among platelets from cultures. (**C**) Top 10 pathways of differentially expressed genes in platelets from MK-MSC cocultures compared with MKs cultured alone. (**D**) Gene expression related to platelet aggregation in platelets from MK-MSC cocultures compared with MKs cultured alone.

Following coculture of wild-type murine MKs with wild-type MSCs or MKs cultured alone, a transcriptomics analysis was conducted on platelets generated in cultures. A principal component analysis revealed the distribution and clustering between samples within each group (Figure 4A). The differentially expressed genes among platelets from MSC cocultures compared with platelets from MKs cultured alone displayed stark differences between groups (Figure 4B). Differentially expressed genes are listed in Supplemental Table 1. Among the top pathways of differentially expressed genes was mitochondrial organization, ATP metabolic process, mitochondrial transport and cellular respiration (Figure 4C). Additionally, platelets from MSC-MK cocultures were downregulated in numerous genes involved in platelet activation (Figure 4D).

### Reduced levels of mitochondria lead to activation of LYN signaling in platelets.

**Figure 5:**
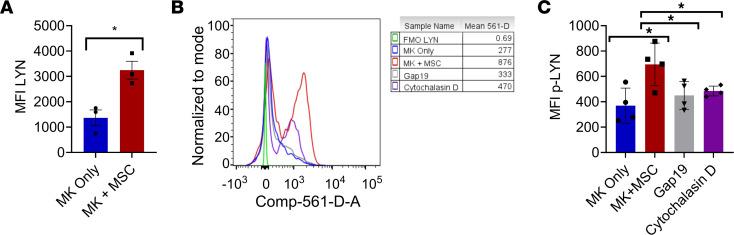
MSCs upregulate LYN signaling in platelets. C57BL/6 murine MKs were cultured together with C57BL/6 MSCs or cultured alone. Platelets from cultures were analyzed by flow cytometry for (**A**) total LYN mean fluorescence intensity (MFI). C57BL/6 murine MKs were cultured together with C57BL/6 MSCs, with Gap19-treated MSCs, cytochalasin D–treated MSCs, or cultured alone. (**B**) Histogram of p-LYN signal among platelets from cocultures. (**C**) MFI p-LYN signal among platelets from cocultures. *P ≤ 0.05. *n* = 3–4 mice. Data in **A** were analyzed with 2-tailed, unpaired Student’s *t* test. Data in **C** were analyzed by 1-way ANOVA with Tukey’s multiple comparison test. Data are presented as mean ± SEM.

Previously it was shown that LYN activation in platelets negatively regulates integrin αIIβ3 signaling (29) and prevents their activation (30). Platelets from MSC-MK cocultures were found to have increased total LYN expression by flow cytometry (Figure 5A) compared with control MK-only cultures. Examination of phosphorylation of LYN (p-LYN) revealed that coculture of MK and MSC led to increased p-LYN signaling compared with MKs cultured alone (Figure 5, B and C, and Supplemental Figure 5, A and B). Treatment of MSCs with Gap19 to inhibit Cx43 prevented LYN signaling activation (Figure 5, B and C, and Supplemental Figure 5, A and B). Tunneling nanotubes are often formed between cells to aid in intracellular organelle trafficking (31). Furthermore, tunneling nanotubes can form by actin polymerization and connect gap junctions between cells (32, 33). MSCs treated with cytochalasin D actin polymerization inhibitor prevented LYN signaling from occurring (Figure 5, B and C, and Supplemental Figure 5, A and B), suggesting that tunneling nanotubes formed between MSCs and MKs are mediating mitochondrial transfer between cells.

### Platelets from both SCD mice and patients with SCD are more glycolytic.

**Figure 6:**
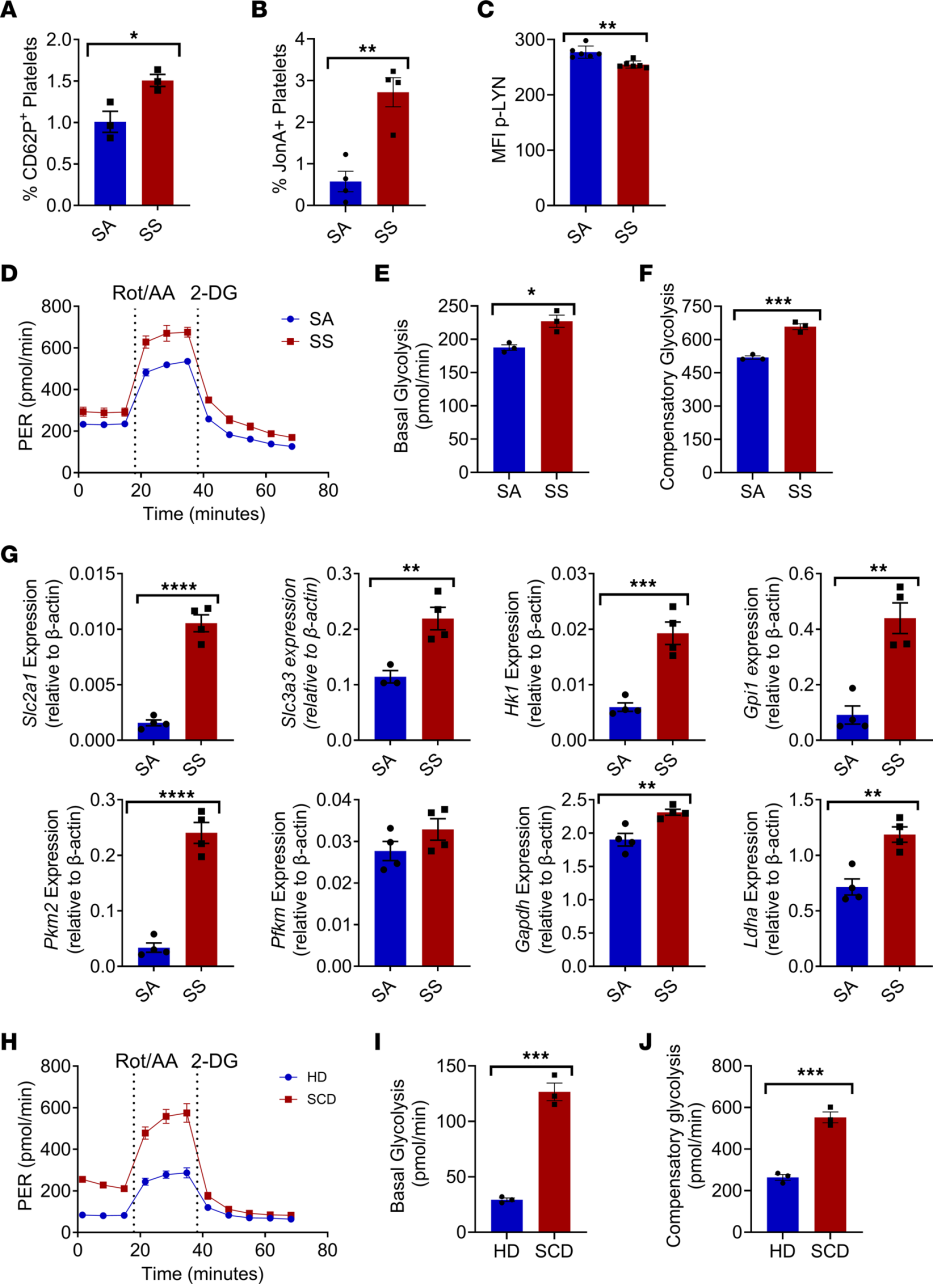
Activation and metabolic profile of platelets from SCD mice. Peripheral blood platelets from the Townes model of SCD or SA control mice were analyzed by flow cytometry for (**A**) CD62P expression (*n* = 3 mice) and (**B**) JonA expression (*N* = 4 mice). (**C**) Quantification of p-LYN expression by mean fluorescence intensity (MFI) (*n* = 6 mice). (**D**) Representative Seahorse glycolytic rate assay conducted on platelets from SCD mice compared with SA control mice (*n* = 3 mice). (**E**) Basal glycolysis levels (*n* = 3 mice). (**F**) Compensatory glycolysis levels (*n* = 3 mice). (**G**) Sorted MKs from SCD mice or SA control mice were analyzed by RT-PCR for glycolysis associated gene expression (*n* = 4 mice). Peripheral blood platelets from SCD or healthy donor patients were analyzed by (**H**) Seahorse glycolytic rate assay (*n* = 3). (**I**) Basal glycolysis levels of SCD platelets or healthy donor control platelets (*n* = 3). (**J**) Compensatory glycolysis of SCD platelets or healthy donor control platelets (*n* = 3). *P ≤ 0.05, **P ≤ 0.01, ***P ≤ 0.001, ****P ≤ 0.0001. Data were analyzed with 2-tailed, unpaired Student’s *t* test. Data are presented as mean ± SEM.

Platelets from the Townes mouse model of SCD were found to have increased CD62P expression (Figure 6A) and JonA expression (Figure 6B) compared with control SA heterozygous mice, confirming previous findings (34). Platelets from SCD mice had reduced activation of p-LYN (Figure 6C and Supplemental Figure 5, C and D) signaling compared with SA mice platelets. Seahorse analysis of platelets from SCD mice revealed increased glycolysis (Figure 6D), including enhanced basal glycolysis (Figure 6E) as well as compensatory glycolysis (Figure 6F), compared with SA control mice. MKs from SCD mice displayed significantly increased expression of numerous genes related to glycolysis compared with SA MKs, including *Slc2a1*, *Slc3a3, Hk1*, *Gpi1*, *Pkm2*, *Gapdh*, and *Ldha* (Figure 6G). Platelets from patients with SCD also displayed similarly glycolytic profiles (Figure 6, H–J). We examined the localization of SCD or SA control MKs to *Lepr^+^* MSCs using whole-mount imaging of bone marrow and found that SCD MKs are located at an average distance of 26.1 μm away from the nearest MSC whereas SA MKs are located 21.0 μm away from the nearest MSC (Supplemental Figure 6).

### Impaired MSCs in SCD contribute to increased platelet activation.

**Figure 7:**
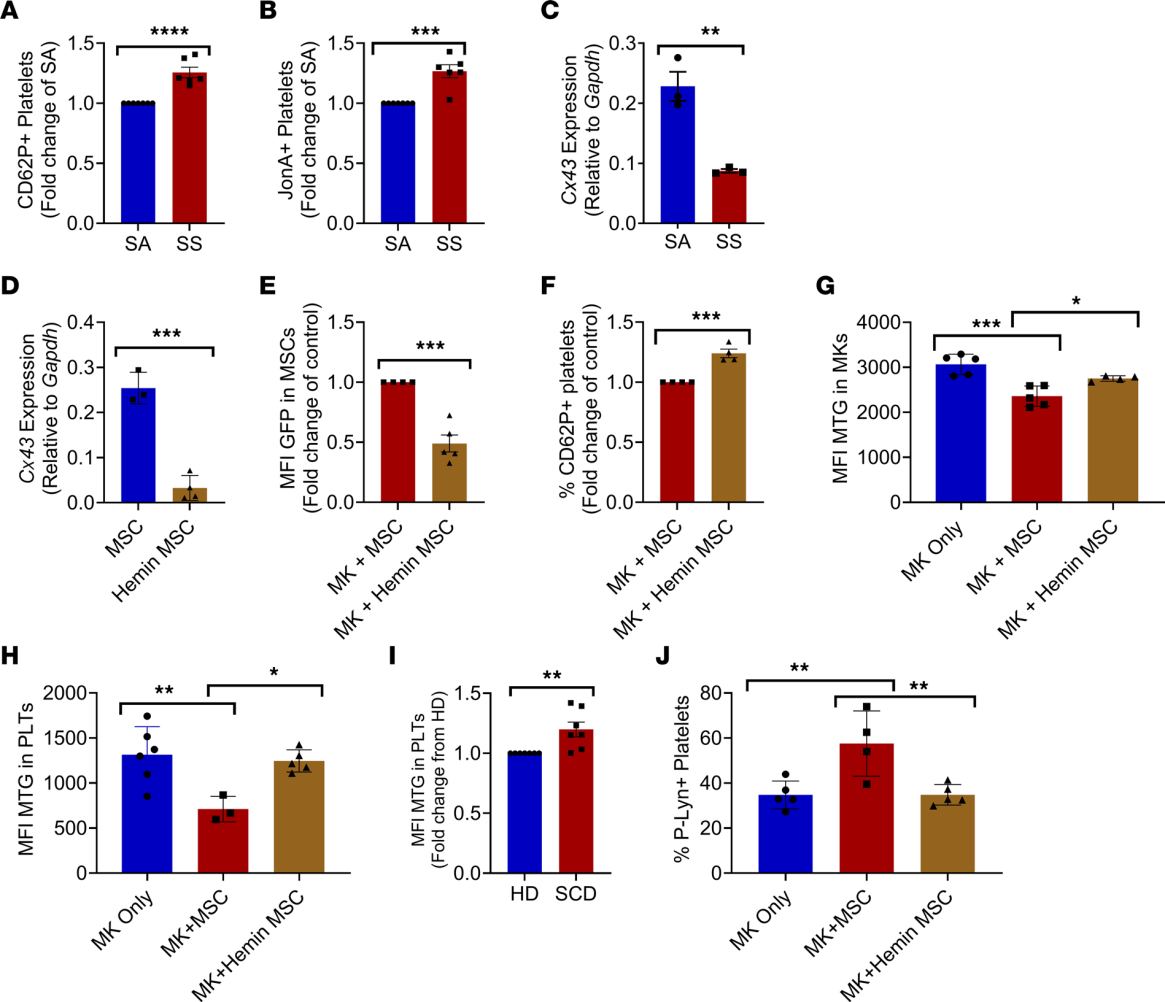
MSCs contribute to increased platelet activation in SCD. SA murine MKs were cultured together with MSCs from SS or SA mice. Platelets from cocultures were analyzed by flow cytometry for expression of (**A**) CD62P (*n* = 6) and (**B**) JonA (*n* = 6). SA or SS MSCs were analyzed by RT-PCR for expression of (**C**) *Cx43* (encoded by *Gja1*) relative to *Gapdh* (*n* = 3). (**D**) *Cx43* (encoded by *Gja1*) relative to *Gapdh* in 20 μM hemin-treated MSCs or vehicle control-treated MSCs (*n* = 3–4). *PhAM-floxed;E2a-cre* murine MKs were cultured together with hemin-treated MSCs or vehicle-treated MSCs. Following coculture, flow cytometry assessment of (**E**) mean fluorescence intensity (MFI) of PhAM signal in MSCs (*n* = 4–5). (**F**) Percentage of CD62P^+^ platelets from hemin-treated MSC MK cocultures normalized to the MK + MSC group (*n* = 4). C57BL/6 murine MKs cultured alone or together with vehicle-treated MSCs or hemin-treated MSCs. (**G**) Mitotracker green signal in MKs (*n* = 5) and (**H**) mitotracker green signal in platelets (*n* = 3-6). (**I**) Mitotracker green signal in peripheral blood platelets from patients with SCD or healthy donors (*n* = 7). (**J**) Flow cytometry analysis of P-LYN expression in murine platelets from cocultures of MKs only, MKs + MSCs, and MKs + hemin-pretreated MSCs (*n* = 4–5). *P ≤ 0.05, **P ≤ 0.01, ***P ≤ 0.001, ****P ≤ 0.0001. Data in **A**–**F** and **I** were analyzed with 2-tailed, unpaired Student’s *t* test. Data in **G**, **H**, and **J** were analyzed by 1-way ANOVA with Tukey’s multiple comparison test. Data are presented as mean ± SEM.

Given the important role of MSCs for maintaining low platelet activation at steady state (1), we were interested to investigate whether SCD MSCs, which have decreased functionality in SCD, have alterations in their ability to modulate platelet activation. Interestingly, MSCs from SCD mice cocultured with healthy MKs produced platelets with significantly increased expression of CD62P (Figure 7A) and JonA (Figure 7B) compared with platelets produced by MKs cultured with control SA MSCs. SCD mice have reduced Cx43 gene expression (Figure 7C) and protein levels (Supplemental Figure 7) compared with SA control mice. SCD leads to abundant levels of free heme in the circulation (35). Coculture of human cord blood–derived MKs with MSCs from patients with SCD led to reduced mitochondrial uptake compared with coculture with MSCs from healthy donors (Supplemental Figure 8 and Supplemental Videos 3–6). To examine the role of hemin on MSC Cx43 expression and ability to uptake MK-derived mitochondria, we treated MSCs with 20 μM hemin and found a significant reduction in Cx43 expression by RT-PCR (Figure 7D). Coculture of MKs from *PhAM-floxed;E2a-cre mice* with hemin-treated MSCs led to a significant inhibition in their ability to uptake mitochondria from MKs (Figure 7E and human cocultures in Supplemental Video 6), which resulted in increased CD62P^+^ activation of platelets (Figure 7F). MKs cocultured with heme-treated MSCs displayed increased mitochondrial retention compared with wild-type MSC cocultures (Figure 7G), along with platelets from cocultures (Figure 7H) and consistent with *Cx43-floxed* MSC-MK cocultures (Figure 3, A and B, and Supplemental Video 7). MKs following coculture with heme-treated MSCs displayed increased glucose uptake ability compared with cocultures with wild-type MSCs (Supplemental Figure 1I) and reduced intracellular ATP/ADP ratios (Supplemental Figure 1J). Platelets from patients with SCD also showed increased mitochondrial retention compared with healthy donor control platelets (Figure 7I). Examination of p-LYN activation in platelets revealed that hemin-treated MSCs cocultured with MKs prevented p-LYN activation in platelets compared with control MSC cocultures (Figure 7J) and, similar to the effect of Cx43, inhibited MSCs (Figure 5C). Importantly, wild-type mice treated with hemin for 2 weeks phenocopied our in vitro cocultures, leading to reduced Cx43 gene and protein expression in MSCs (Supplemental Figure 9, A and B), reduced mitochondrial uptake by MSCs (Supplemental Figure 9C), increased MK mitochondrial retention (Supplemental Figure 9D), and increased expression of several glycolysis genes in MKs (Supplemental Figure 9E) compared with control mice. Platelets in heme-treated mice also displayed increased glycolysis (Supplemental Figure 9, F–H) relative to control mice.

## Discussion

To our knowledge, in this study, we uncover for the first time the ability of MKs to transfer mitochondria to MSCs, leading to the formation of platelets with low baseline activation levels. Reduction of mitochondria numbers in MKs leads to platelets that are less glycolytic and activates LYN signaling. While it was previously demonstrated that activated platelets can transfer mitochondria to MSCs (2), here we demonstrate a mechanism used by MKs to produce platelets at a low activation state. This further expands on the complexity of the bone marrow niche (36) and raises the possibility that MSCs have additional regulatory roles not just for hematopoietic stem cells but also for mature blood cells, which remain to be explored. Protocols for expanding platelets in vitro for transfusion require the production of resting platelets (37), and the inclusion of MSCs during expansion could be of direct benefit.

We found that MKs are adept at transferring mitochondria to MSCs, whereas MSCs transfer minimal amounts of mitochondria to MKs. What causes MKs to preferentially transfer mitochondria to MSCs remains to be further investigated. Mitochondrial motor protein kinesin requires ATP hydrolysis to direct movement (38), and interestingly, we found that MKs from MSC-MK cocultures have increased ATP/ADP ratios compared with MKs cultured alone. It is possible that MSCs use alternative forms of communication, such as microvesicles, to also impart changes to MK energetic states. Distance between MSCs and MKs appears to be a factor in mediating their communication as MSC-specific Cx43-KO mice showed an increase in MK localization away from MSCs compared with wild-type mice. Furthermore, we found that cytochalasin D actin inhibitor treatment during MSC-MK coculture prevented mitochondrial transfer between the two, suggesting a potential role of tunneling nanotubes mediating the transfer of mitochondria. SCD MKs were also localized further from SCD MSCs compared with SA MSC-MK localization, possibly due to the abnormal vascular networks present in SCD bone marrow (39).

Hemolysis in SCD leads to thrombosis and inflammation and promotes complications such as stroke, leukocyte adhesion, and vascular activation (40). A direct effect of free heme on platelets leads to their activation following binding of CLEC-2 on their surface (41, 42). Our study has uncovered an indirect effect of heme on platelet activation occurring at the thrombogenesis stage through SCD MSC interactions with MKs. Specifically, we showed that SCD MSCs without the addition of heme promoted enhanced platelet activation compared with SA MSC cultures. Downregulation of Cx43 in SCD MSCs, phenocopied by heme-treated healthy MSCs, prevented mitochondrial transfer from MKs to MSCs leading to increased activation of platelets produced from cultured MKs. In vivo heme treatment of mice confirmed our in vitro studies and led to a similar reduction of MK mitochondrial transfer and MSC mitochondrial uptake. Platelet functionality was also altered, as heme treatment or Cx43 inhibition in MSCs led to a reduced platelet aggregation ability, potentially contributing to the heightened bleeding risk that occurs in patients with SCD (43). Our findings further highlight the importance of the bone marrow microenvironment for contributing to SCD pathophysiology and the need for novel strategies to improve the bone marrow niche in SCD. In support of this, we previously showed that transfusions can correct MSC dysfunction (21) and can lead to reversal of a disorganized vascular network in SCD (39). Although the platelet activation state was not interrogated following transfusions (21), improvements in MSC functionality following transfusions may also translate into enhanced MSC ability to regulate platelet activation. Given the high levels of oxidative stress in the bone marrow microenvironment (21, 39), therapeutics that can lower oxidative stress such as the antioxidant N-acetyl cysteine or methods for improving hemoglobin oxygen affinity such as voxelotor can similarly improve MSC functionality (21, 44) and may also indirectly lead to improved regulation of platelet activation. While genetic reprogramming of stem cells for gene therapy may directly lead to MKs producing platelets with a lower activation state in SCD, improving the bone marrow microenvironment may also impart changes to platelet activation.

We found that platelets from SCD mice and patients with SCD displayed a glycolytic phenotype, which has been associated with increased platelet activation (45, 46). Previous studies found that humans with SCD have reduced mitochondrial complex V function, leading to reduced mitochondrial respiration (34). Interestingly, impaired mitochondrial complex V functionality has been correlated with increased activation of glycolysis (34, 47, 48). Approaches for metabolically targeting cancer cells to inhibit cancer progression (49) by disrupting various transporters or metabolic enzymes are actively being investigated and could potentially be repurposed to lower glycolysis in SCD MKs or platelets, leading to reduced platelet activation. In addition to SCD, our findings may have implications for drug development to target metabolic disorders, diabetes, and obesity-driven thrombosis as a novel mechanism to lower platelet activation.

## Methods

### Sex as a biological variable

Both male and female mice and human samples from patients with SCD or healthy donors were used throughout the study. Mice for experimental and control groups were age and sex matched for individual experiments. Similar findings were reported for both sexes.

### Mice

The Townes model of SCD was used in this study (50). These mice have severe organ damage, including enlarged spleens, liver necrosis, and vascular occlusion in the kidneys (51). SA sickle trait mice were used for controls since they are genetically similar to SS mice but do not carry any disease related abnormalities. Mice used ranged between 8 and 12 weeks of age. C57BL/6J, B6;129-Hbb^tm2(HBG1,HBB*)Tow^/Hbb^tm3(HBG1,HBB)Tow^ Hba^tm1(HBA)Tow^/J (Townes), B6.129(Cg)-Lepr^tm2(cre)Rck^/J (Lepr-cre), B6.129S7-Gja1^tm1Dlg^/J (Cx43-floxed), B6;129S-Gt(ROSA)26Sor^tm1(CAG-COX8A/Dendra2)Dcc^/J (PhAM-floxed), and B6.FVB-Tg(EIIa-cre)C5379Lmgd/J mice were purchased from Jackson Laboratory and bred in-house. All mice were housed in a pathogen-free facility at 22°C using a 12:12-hour-light/dark cycle.

### Human samples

SCD blood or bone marrow samples were obtained from patients (*n* = 7, median age 16 years, age range 10–34 years, 3 patients were female and 4 were male) who were at steady state without any SCD complications. Four patients were on a chronic red cell exchange therapy (every 3–4 weeks for at least 2 years using leukodepleted units, phenotype matched for the C, E, and K red cell antigens), and blood samples were drawn just prior to transfusion. One patient was on voxelotor, and 2 were on crizanlizumab treatment. Cord blood units were obtained from the National Cord Blood Program of the New York Blood Center. Cord blood CD34^+^ cell differentiated MKs were derived using our prior methods (1). Human healthy donor bone marrow MSCs were purchased from AllCells.

### In vitro culture experiments

Murine bone marrow MSCs were sorted using our previous methods (21) based on the following surface markers: CD45^–^ (Biolegend, catalog 157614), Ter119^–^ (Biolegend, catalog 116220), CD31^–^ (Biolegend, catalog 102528), CD51^+^ (Biolegend, catalog 104106), and PDGFRα^+^ (Biolegend, catalog 135914). For coculture experiments, murine MSCs were allowed to adhere for 24 hours following sorting. Primary murine MK enriched cells were obtained from flushed bone marrow using CD41 biotin (Biolegend, catalog 133930) bound to streptavidin-labeled magnetic beads (Miltenyi, catalog 130-048-101) followed by enrichment with Large Cell Columns (Miltenyi). 200,000–350,000 MK enriched cells were cocultured together with 50,000–75,000 sorted murine MSCs in 24-well plates for 18 hours in media consisting of Stemspan (Stemcell Technologies) supplemented with Penicillin/Streptomycin (Fisher), nonessential amino acids (Fisher), and murine TPO (50 ng/mL; R&D Systems) followed by analysis with flow cytometry, imaging, or RT-PCR. Following coculture, MKs and platelets in suspension were manually separated from MSCs, and MSCs were washed twice with PBS to ensure complete removal of any loosely bound cells. For MSC pretreatment before coculture, MSCs were treated with 20 μM hemin (Frontier Scientific) or vehicle control for 8 hours using our established methods (52) and washed twice to ensure complete hemin removal. For Gap19-treated cultures, MSCs were pretreated with 100 μM Gap19 (Tocris) (53) before coculture, and control MSCs were treated with a scrambled control peptide for 8 hours followed by coculture. To inhibit tunneling nanotube formation, certain cocultures were treated with 1 μM cytochalasin D (Tocris) (54). Select groups of MKs were treated with 1 μM Oligomycin A (MilliporeSigma) or vehicle control for 1 hour prior to coculture. The ATP/ADP ratio of cells was analyzed with the EnzyLight assay kit (Bioassay Systems) using 10,000 cells per well and normalized to protein content by BCA assay (Abcam). For human cocultures, 50,000 MSCs were cultured together with 50,000 cord blood–derived MKs on day 12 of differentiation for 18 hours in media consisting of Stemspan (Stemcell Technologies) supplemented with penicillin/streptomycin (Fisher), nonessential amino acids (Fisher), and human TPO (50 ng/mL; R&D Systems).

### Flow cytometry analysis

Murine bone marrow MSCs were isolated using previous protocols (21). Red blood cells were lysed from bone marrow cells using the ammonium chloride–based solution Pharm Lyse Buffer (BD). Flow cytometry experiments were performed using a LSRFortessa (BD). Cells were sorted on FACSAria III (BD) Cell Sorter, and data were analyzed using FlowJo software. Debris were excluded based on forward scatter and side scatter profiles, whereas dead nucleated cells were excluded with a nuclear exclusion stain such as DAPI (MilliporeSigma). For analysis of intracellular staining, following surface marker staining, cells were fixed and permeabilized using 1% PFA and Triton X-100, respectively. Human and murine platelets were analyzed by gating on platelet-sized particles using whole blood samples as a reference. Isotype controls were used to evaluate antibody specificity and determine gating. Chemical stain for mitotracker green was purchased from Fisher Scientific and used according to manufacturer protocols. In select cocultures, MKs were labeled with PKmito Deep Red (Cytoskeleton Inc.) or Mito Live Orange (Abberior) prior to coculture with MSCs to track mitochondrial transfer. Antibody for measurement of p-LYN activation was purchased from Cell Signaling Technology (catalog 94361S). Glucose uptake was performed using 50 μg/mL 2-NBDG, incubated at 37°C for 30 minutes (Abcam).

### ImageStream flow cytometry

To examine mitochondria transfer in transplanted mice, 1 femur and 1 tibia were isolated from each mouse. To examine bone marrow MKs, flushed marrow was depleted for Ter119^+^ cells using biotinylated antibodies (Biolegend, catalog 116204) followed by streptavidin-bound magnetic beads (Miltenyi, catalog 130-048-101), and then stained for CD41 PE (Biolegend, catalog 133906). Cells were washed and resuspended in 50 μL DAPI solution. For stromal cell analysis, 1 tibia and 1 femur were digested using our prior methods (21) with 1 mg/mL collagenase IV and 2 mg/mL dispase in PBS (MilliporeSigma) for 40 minutes. Stromal cells were enriched by depletion of CD45^+^ (Biolegend, catalog 103104), Ter119^+^ (Biolegend, catalog 116204), and CD31^+^ (Biolegend, catalog 102404) cells using biotinylated antibodies followed by streptavidin-bound magnetic beads (Miltenyi). Enriched bone marrow was stained with CD45 Alexa Fluor 700 (Biolegend, catalog 103128), Ter119 Alexa Fluor 700 (Biolegend, catalog 116220), CD31 Alexa Fluor 700 (Biolegend, catalog 102444), CD51 PE (Biolegend catalog, 104106), and CD140a Percp-Cy5.5 (Biolegend, catalog 135914). The cells were washed and resuspended with 50 μL DAPI solution. Cells were analyzed using an Amnis ImageStream Mark II instrument at ×60 magnification. IDEAS software was used for analysis.

### Hematopoietic transplantations

*PhAM-floxed;E2a-cre* or B6 mice were lethally irradiated with 12 Gy, using 2 split doses, 4 hours apart using an X-RAD 300. Four hours following the last dose, mice were injected i.v. with 2 million bone marrow mononuclear cells. Mice were treated with antibiotics for 2 weeks in their drinking water following transplant. Mice were harvested for analysis at 12 weeks after transplant for analysis.

### Seahorse analysis

Platelet-rich plasma was isolated from whole blood using our prior methods (1). Platelets were pelleted and washed once with PBS followed by quantification by Advia hematology analyzer. Platelets were spun again at 2,500*g* and resuspended in prewarmed assay media (unbuffered DMEM supplemented with 1 mM pyruvate, 2 mM glutamine, and 20 mM glucose for murine platelets or 5.5 mM glucose for human platelets; Agilent). 50 μL platelet solution was loaded into each well of an XFp microplate previously coated with 1.12 μg Cell-Tak (Corning) for a final count of 40 × 10^6^ platelets/well for murine platelets or 18 × 10^6^ platelets/well for human platelets. The cell plate was subsequently centrifuged (1,200*g* for 3 minutes with no brake) to form a monolayer in the well. 130 μL Seahorse assay media was added to each well for a final volume of 180 μL. The cell plate was placed in a non-CO_2_ incubator at 37°C for 30 minutes before the assay. For glycolytic rate assay measurement, platelets were consecutively treated with rotenone/antimycin A (0.5 μM) and 2-deoxy-D-glucose (50 mM). For MK analysis, MKs collected from cocultures were spun at 200*g* to separate MKs from platelets. MKs were spun again and resuspended in prewarmed assay media (unbuffered DMEM supplemented with 1 mM pyruvate, 2 mM glutamine, and 20 mM glucose; Agilent). 50 μL MKs (2 × 10^5^ to 3.5 × 10^5^ cells per well) were loaded into each well of an XFp microplate previously coated with 1.12 μg Cell-Tak (Corning). The cell plate was subsequently centrifuged (300*g* for 3 minutes with no brake) to form a monolayer in the well. 130 μL Seahorse assay media was added to each well for a final volume of 180 μL. The cell plate was placed in a non-CO_2_ incubator at 37°C for 30 minutes before the assay. For glycolytic rate assay measurement, MKs were consecutively treated with rotenone/antimycin A (0.5 μM) and 2-deoxy-D-glucose (50 mM).

### RT-PCR

For RT-PCR analysis, following cell lysis, mRNA isolation was conducted with the Dynabeads mRNA Direct kit (Fisher). Reverse transcription to produce cDNA was conducted with RNA to cDNA Ecodry Premix (Takara). SYBR Green (ROCHE) was used for performing quantitative PCR. Analysis was done on the ViiA 7 Real-Time PCR System (Applied Biosystems). The comparative Ct method was used to determine relative gene expression and normalized to the reference genes of either *Gapdh* or *β-actin*.

### Confocal microscopy

For sternum imaging, mice were i.v. labeled with CD31 (Biolegend, catalog 102416)/CD144 (Biolegend, catalog 138108) and harvested after 10 minutes. Sternum sections were fixed with 4% PFA for 30 minutes, washed with PBS, and blocked/permeabilized with 20% donkey serum and 0.5% Triton X-100. Tissues were stained with CD41 (Biolegend, catalog 133908) and Lepr (R&D Systems, catalog AF497) and subsequent secondary antibody staining of goat anti-donkey Alexa Fluor 568 (Fisher, catalog A-11057). Whole-mount stained tissues were imaged on the Zeiss LSM 800 microscope. Zen Blue Software (Zeiss) was used to manually quantify the distances from each MK to the nearest located MSC. For live-cell mitochondrial imaging and tracking, primary *PhAM-floxed;E2A-cre*^+^ murine MKs were cocultured with a confluent monolayer of C57BL/6 MSCs. Human cord blood CD34^+^ cell–derived MKs were prelabeled with Live Orange Mito (Abberior) fluorescent probe and cocultured with bone marrow MSCs from healthy donors either unlabeled or labeled with PKmito Deep Red (Cytoskeleton Inc). Confocal microscopy was performed in the Rockefeller University’s BioImaging Resource Center (RRID:SCR_017791). Time-lapse confocal images were acquired every 30 seconds for 1–3 hours. Images were acquired on a Zeiss LSM 980 microscope with Zen 3.6 software and a ×63 1.4NA oil objective using 561 nm and 639 nm laser lines. Images, acquired as “.czi” file format, were iteratively deconvolved using Huygens Professional Software (Scientific Volume Imaging) to reduce background noise and enhance the fluorescent signal and exported as “ICS2” files for further analysis using Imaris software (Oxford Instruments). Track speed, number of mitochondria per time point, volume, and track displacement parameters were derived by applying filamentous surface renderings matched to the shape of the mitochondria within the images.

### Platelet aggregation studies

#### Light aggregometry.

Washed platelets in Hepes/Tyrode’s buffer were prepared from whole blood collected from the retro-orbital plexus of age- and sex-matched *Lepr-cre;Cx43-floxed* mice or wild-type control mice using our prior methods (55). Platelet suspensions (320 × 10^6^ /mL) were analyzed in response to 1 U/mL Thrombin (MilliporeSigma) or 1 μg/mL CRP (Pplus Products) using Pap8E lumi-aggregometer (Bio/Data). Platelet aggregation was quantified over time as a function of light transmission.

#### Flow cytometry.

A flow cytometry–based aggregation assay was conducted on cultured platelets (27). Platelets from cocultures were collected and split into two. Half of the sample was labeled with CD42b Alexa Fluor 647 (Biolegend, catalog 303924), and half of the sample was labeled with CD42b PE (Biolegend, catalog 303906), washed twice, and then combined. Platelets were activated with 1 U/mL Thrombin for 30 minutes and subsequently analyzed by flow cytometry for the presence of aggregates positive for both PE and Alexa Fluor 647 fluorescent signals. For general platelet activation studies, platelets were activated with 1 U/mL Thrombin or 0.1 μg/mL CRP for 30 minutes and subsequently analyzed by flow cytometry.

### Measurement of thrombus formation by T-TAS

T-TAS is an automated microfluidic based system designed to examine platelet thrombus formation under continuous perfusion (56, 57). Whole blood collected in 3.2% sodium citrate from *Lepr-Cre;Cx43-floxed* mice or control mice was subjected to the atheroma chip assay in the T-TAS -Plus System (Zacros). The microfluidic chip was coated with type I collagen and tissue thromboplastin, which activate the coagulation system when the platelets were perfused under shear stress.

### Western blot analysis

For Western blot analysis, cells were lysed in cold 1× RIPA buffer (Millipore) in the presence of 1× Protease Inhibitor Cocktail (MilliporeSigma). Protein concentration was determined using a Pierce BCA protein assay kit (Thermo Fisher Scientific). Equal amounts of proteins were loaded on to polyacrylamide gels. Antibodies against CX43 (Fisher, catalog BS-0651R), LYN (Fisher, catalog BS-2906R), p-LYN (Cell Signaling Technology, catalog 70926S), and β-actin (Cell Signaling Technology, catalog 4970S) were used. Quantification of the Western blot was performed with ImageJ (NIH).

### RNA-Seq analysis

Wild-type murine primary MKs were cocultured together with wild-type murine bone marrow MSCs or cultured alone as a control. Platelets generated in vitro were pooled together from 5 wells to generate individual samples for analysis. RNA was extracted using a NucleoSpin RNA-XS kit (Takara). Library preparation, RNA-Seq analysis, and bioinformatics analysis were conducted with the Applied Bioinformatics Laboratory at the NYU Grossman School of Medicine. Upon ensuring sample quality with an Agilent bioanalyzer, libraries were prepared with the low input Smart-Seq HT kit (Takara) followed by paired-end analysis on the SP 100 Illumina NovaSeq 6000 machine. Low-quality reads were removed and filtered by fastp. Filtered reads were quantified by Kallisto using the mm10 transcriptome index (58). Pairwise comparison of two groups was performed by DESeq2 (59), and the batch effect was included in the design of DESeq2. Cutoffs of fold change ≥2, adjusted *P* ≤ 0.05, and ≥1 transcript per million in at least 1 group were adopted to identify differentially expressed genes in the pairwise comparisons. Log-transformed normalized counts were used in principal component analysis. Gene ontology and enrichment analysis were applied by clusterProfiler (60).

### In vivo hemin treatment

To mimic long-term hemolysis, C57BL/6 mice were injected i.v. with hemin (Frontier Scientific) or PBS (control) 3 times per week for 2 weeks at a dose of 50 μmol/kg.

### Statistics

We used an unpaired 2-tailed Student’s *t* test to determine significance for normal data distributions and assumed both populations have the same standard deviation. An α level of 0.05 was used to determine statistical significance. For multiple sample comparison, a 1-way ANOVA was done using Tukey’s post hoc test for multiple comparisons correction or repeated measure correction. For distance distributions, a 2-sample Kolmogorov-Sminov test was used. All murine data comprised at least 3 repeated experiments. *P* values of less than 0.05 were considered statistically significant.

### Study approval

All murine experiments were approved by the Animal Use and Care Committee of New York Blood Center. All human studies were approved by the Institutional Review Boards of the New York Blood Center and Montefiore Medical Center. Informed consent was obtained from all patients prior to their participation in the study.

### Data availability

Data supporting the findings of this study are available from the corresponding author upon request. RNA-Seq data have been deposited in Gene Expression Omnibus (GEO) under the acquisition number GSE262752. Values for all data points in graphs are reported in the Supporting Data Values file.

## Author contributions

CG assisted with Seahorse, RNA-Seq, and bioinformatics analysis as well as Western blot and stromal cultures. YD and PA Spezza conducted genomic analysis, cocultures, flow cytometry, and data analysis. PB and GR assisted with confocal microscopy and flow cytometry. AT performed transplants and Seahorse, coculture, and data analysis. AG assisted with data analysis and statistical analysis. YL helped with sickle mouse experiments. BS conducted platelet light aggregometry analysis. WBM and DM were involved with all aspects of selection, recruitment, and provision of blood samples from patients and controls. AM conceived the study and wrote the manuscript. XA, CAL, PA Shi, HZ, and KY assisted with experiment planning and data analysis. All authors helped to edit the manuscript.

## Supplementary Material

Supplemental data

Unedited blot and gel images

Supplemental table 1

Supplemental video 1

Supplemental video 2

Supplemental video 3

Supplemental video 4

Supplemental video 5

Supplemental video 6

Supplemental video 7

Supporting data values

## Version 1 02/27/2025

In-Press Preview

## Version 2 04/15/2025

Electronic publication
