# Supplementary material for: Megakaryocytes transfer mitochondria to bone marrow mesenchymal stromal cells to lower platelet activation
Source: J Clin Invest. 2025 Feb 27;135(8):e189801. doi: 10.1172/JCI189801 (PMC11996913; doi:10.1172/JCI189801)
Supplement: Supplemental data [file jci-135-189801-s021.pdf]

Supplementary Figures

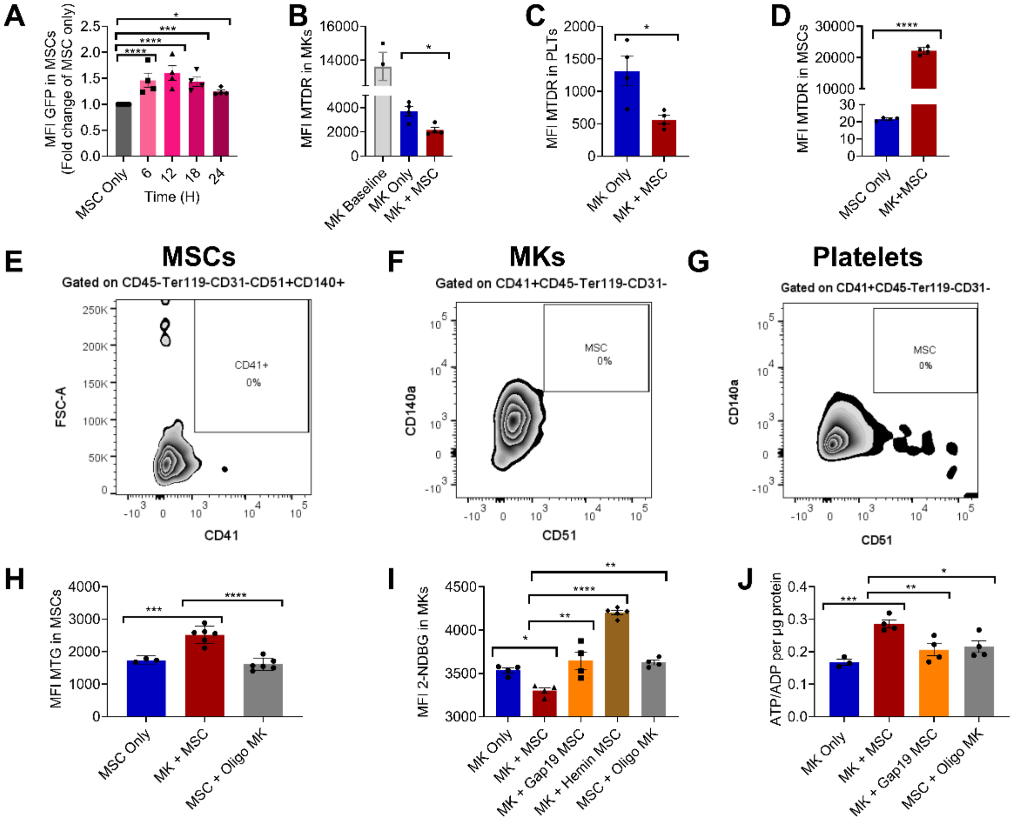

Supplemental Figure 1. Mitochondrial changes in murine MSCs and metabolic changes in MKs following murine MSC-MK co-cultures. Wild-type murine MSCs were cultured with *PhAM floxed;E2a-cre+* murine MKs or cultured alone. **A)** MSCs were analyzed by flow cytometry at various timepoints for MFI of PhAM GFP+ signal. C57BL/6 murine MKs were pre-labeled with PKmito Deep Red stain (MTDR) followed by co-culture with C57BL/6 MSCs and compared to MSCs cultured alone or MKs cultured alone. Flow cytometry of MTDR MFI in **B)** MKs before co-culture (MK Baseline), after co-culture (MK+MSC) or cultured alone (MK only), **C)** platelets from co-cultures or MKs cultured alone, and **D)** MSCs from co-cultures or MSCs cultured alone (N=3-4). Following wild-type MK-MSC co-culture, MKs and MSCs were collected and stained for CD41 and MSC surface markers (CD45, Ter119, CD31, CD140a, CD51). **E)** Flow cytometry of MSCs demonstrating no presence of MKs. **F)** Flow cytometry of MKs demonstrating no presence of MSCs. **G)** Flow cytometry of platelets demonstrating no presence of MSCs. C57BL/6 MSCs were cultured with C57BL/6 MKs with or without 1μM oligomycin (Oligo) pre-treatment and compared to MSCs cultured alone. **H)** Flow cytometry of mitotracker green MFI in MSCs following co-culture (N=3-6). C57BL/6 MSCs were cultured with C57BL/6 murine MKs or cultured alone. Selective groups of MSCs were pre-treated with 20μM hemin (MK + hemin MSC) or 100μM Gap19 (MK + Gap19 MSC). One group of MKs were pre-treated with oligomycin (MSC + Oligo MK). **I)** Glucose uptake assay by flow cytometry of 2-NDBG MFI in MKs (N=4-5). **J)** ATP/ADP ratio in MKs from co-cultures normalized to μg protein (N=3-4). * $P \leq 0.05$ ** $P \leq 0.01$ *** $P \leq 0.001$ **** $P \leq 0.0001$. Data in B-D were analyzed with 2-tailed, unpaired Student's t-test. Data in A, H-J were analyzed by 1-way ANOVA with Tukey multiple comparison test. Data shows mean ± SEM.

Supplemental Table 1. Differentially expressed gene list among platelets from MSC co-cultures. C57BL/6 murine MKs were cultured together with C57BL/6 MSCs or cultured alone. Platelets from MKs cultured alone (PLTs_only) or MSC co-cultures (PLTs_MSCs) were pooled for bulk-RNA seq analysis (N=4 per group).

Supplemental Video 1. Mitochondrial transfer from murine MKs to MSCs. MKs from *PhAM-floxed;E2a-cre* mice were cultured together with C57BL/6 MSCs. Images were acquired by confocal microscopy every 30 seconds for 30 intervals. PhAM signal is depicted in white overlayed onto brightfield.

Supplemental Video 2. Mitochondrial transfer from murine MKs to MSCs. Murine MKs from *PhAM-floxed;E2a-cre* mice were cultured together with C57BL/6 MSCs. Following photoconversion of mitochondria within a MK using the UV laser, mitochondria within the MK change from green to red color. Sequential images were acquired by confocal microscopy every 30 seconds for 40 intervals. Transfer of red photoconverted mitochondria from MK to MSC can be observed. Mitochondria are highlighted with a yellow circle using the spot detection tool in Imaris to track mitochondrial transfer.

Supplemental Video 3. Close up view of mitochondrial transfer from human MKs to healthy donor MSCs. Human umbilical cord blood derived MKs were pre-labeled with Mito Live Orange mitochondrial dye followed by co-culture with unlabeled healthy donor MSCs. Images were acquired by confocal microscopy every 30 seconds for 363 intervals. Panels depict XY, XZ and YZ views. Note: this video is a focused region of Supplemental Video 3B, which displays the full field of view. Scale = 25μm.

Supplemental Video 4. Mitochondrial transfer from human MKs to healthy donor MSCs. Human umbilical cord blood derived MKs were pre-labeled with Mito Live Orange mitochondrial dye followed by co-culture with unlabeled healthy donor MSCs. Images were acquired by confocal microscopy every 30 seconds for 363 intervals. Panels depict XY, XZ and YZ views. Note: this is the full field of view, which includes the region highlighted in Supplemental Video 3A.

Supplemental Video 5. Mitochondrial transfer from human MKs to MSCs from a patient with Sickle Cell Disease. Human umbilical cord blood derived MKs were pre-labeled with Mito Live Orange mitochondrial dye followed by co-culture with unlabeled MSCs from a patient with Sickle Cell Disease. Images were acquired by confocal microscopy every 30 seconds for 251 intervals.

Supplemental Video 6. Mitochondrial transfer from human MKs to heme treated MSCs. Human umbilical cord blood derived MKs were pre-labeled with Mito Live Orange mitochondrial dye followed by co-culture with unlabeled healthy donor MSCs that were pre-treated with 20μM hemin for 8 hours. Images were acquired by confocal microscopy every 30 seconds for 251 intervals.

Supplemental Video 7. Mitochondrial transfer from human MKs to MSCs with inhibition of CX43. Human umbilical cord blood derived MKs were pre-labeled with Mito Live Orange mitochondrial dye followed by co-culture with unlabeled MSCs from a healthy donor that were pre-treated with 100μM Gap19 for 8 hours. Images were acquired by confocal microscopy every 30 seconds for 201 intervals.

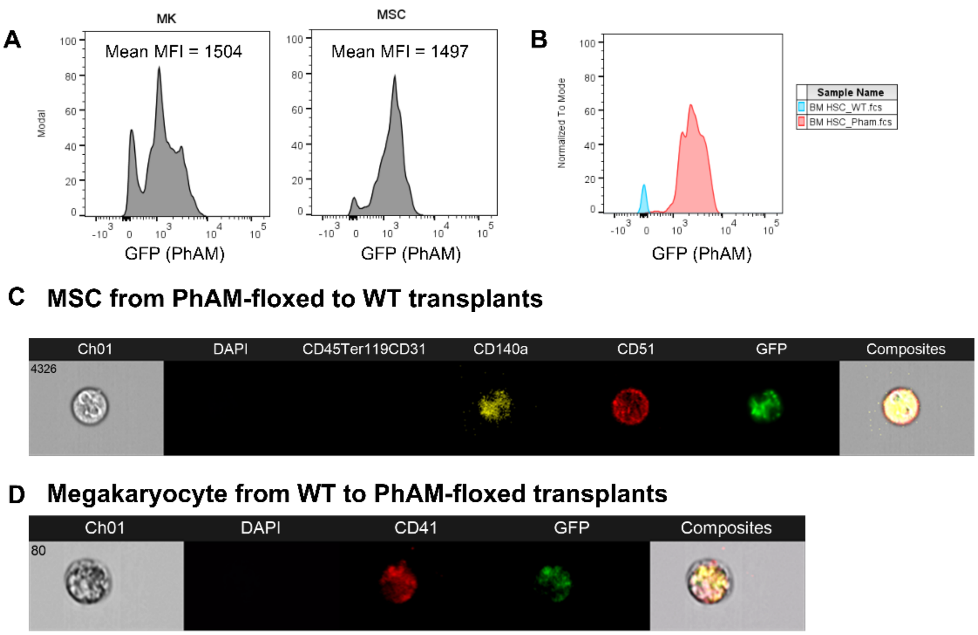

Supplemental Figure 2. Megakaryocytes and MSCs from PhAM floxed mice before and after transplant. A) Mean fluorescence intensity (MFI) of GFP (PhAM) signal in MKs and MSCs from *E2a-cre;PhAM-floxed* mice. **B)** Representative histogram of GFP (PhAM signal) of bone marrow HSCs from *E2a-cre;PhAM-floxed* mice relative to bone marrow HSCs of wild-type mice. **C)** Representative images by Imagestream analysis of MSCs from transplants of *E2a-cre; PhAM-floxed* cells to WT mice stained with DAPI, CD45, Ter119, CD31, CD140a and CD51 along with GFP Pham signal. **D)** Representative images by Imagestream analysis of MKs from transplants of WT cells into *E2a-cre; PhAM-floxed mice* stained with DAPI and CD41 along with GFP PhAM signal.

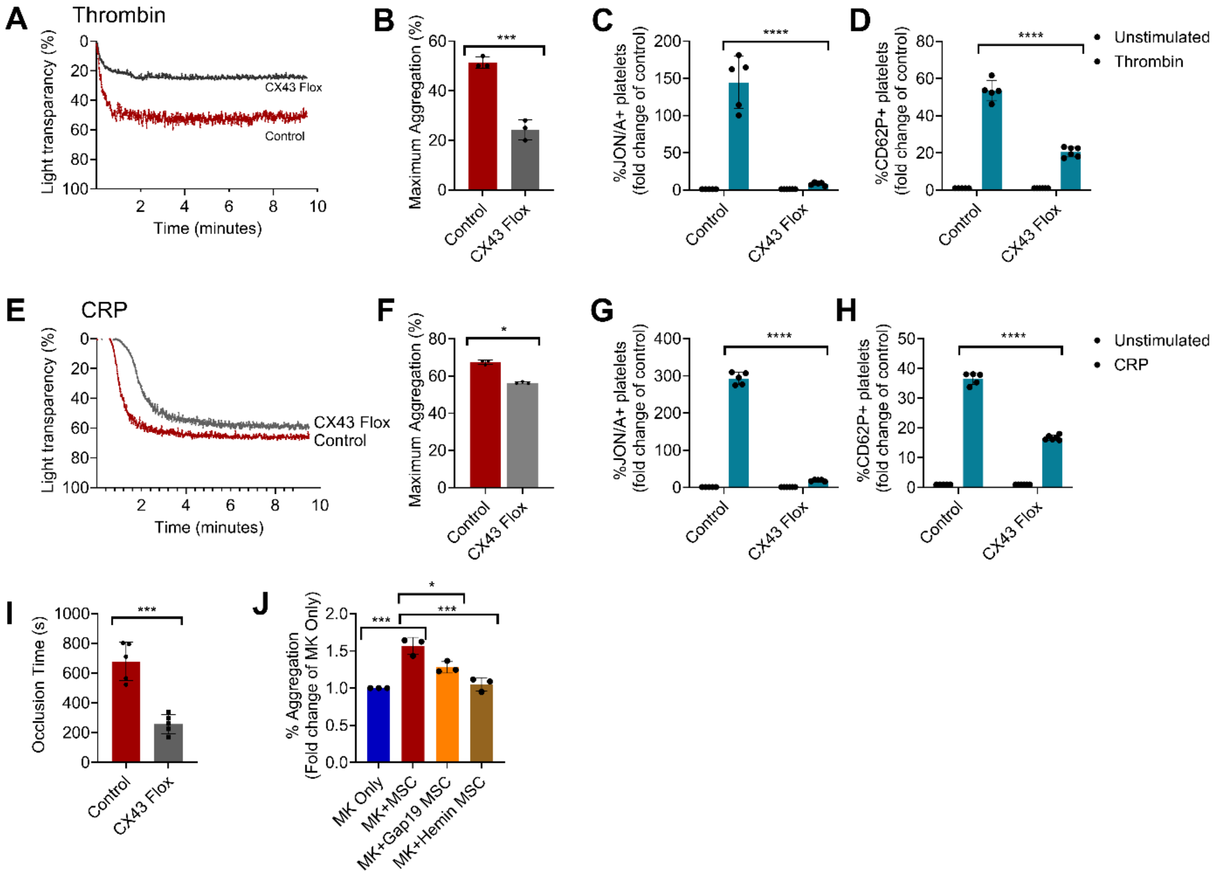

Supplemental Figure 3. *Lepr-cre;Cx43 floxed* mice have impaired platelet aggregation. A) Aggregation of washed platelets from *Lepr-cre;Cx43 floxed* mice and wild-type control mice in response to 1U/mL thrombin. **B)** Quantification of maximum aggregation (%). N=3 mice. *** P≤0.001. Platelet activation in *Lepr-cre;Cx43-floxed* mice or control mice in response to 1U/mL Thrombin by flow cytometry for **C)** %JonA+ platelets and **D)** %CD62P+ platelets (shown as fold change relative to control) (N=5). **E)** Aggregation of washed platelets from *Lepr-cre;Cx43-floxed* mice and wild-type control mice in response to 1µg/mL CRP. **F)** Quantification of maximum aggregation (%). N=3 mice. Platelet activation in *Lepr-cre;Cx43-floxed* mice or control mice in response to 0.1µg/mL CRP by flow cytometry for **G)** %JonA+ platelets and **H)** %CD62P+ platelets (shown as fold change relative to control) (N=5). **I)** Occlusion time (seconds) in *Lepr-cre;Cx43-floxed* mice or control mice from the AR assay on the T-TAS-Plus System, measured in response to collagen activation under shear stress (N=5). Wild-type murine MKs were cultured alone or together with wild-type murine MSCs pre-treated with Gap19, Hemin or control solution. Cultured platelets were subjected to a flow cytometry aggregation assay displaying **J)** % Platelet aggregation in response to 1U/mL thrombin stimulation (represented as fold-change of MK only group) (N=3). * P≤0.05 *** P≤0.001 **** P≤0.0001. Data in B, F and I were analyzed with 2-tailed, unpaired Student's *t*-test. Data in C, D, G, H and J were analyzed by 1-way ANOVA with Tukey multiple comparison test. Data is presented as mean ± SEM.

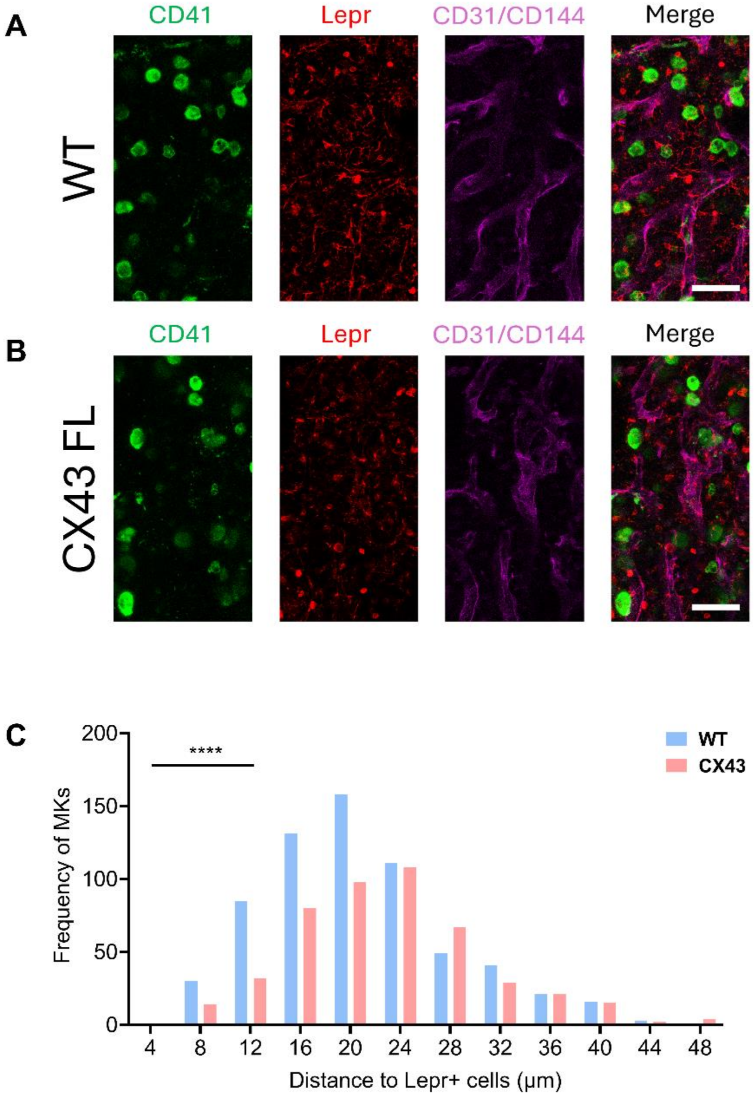

Supplemental Figure 4. Murine megakaryocytes localize away from mesenchymal stem cells with reduced CX43 expression. Whole mount sternum sections stained for CD41, CD31/CD144, and Leptin receptor were imaged by confocal microscopy in **A)** C57BL/6 wild-type (WT) mice or **B)** *Cx43 floxed;Lepr-cre* mice. **C)** Distribution of MKs in relation to sinusoidal Lepr+ MSCs from WT and *Cx43 floxed;Lepr-cre* mice. N = 651 from 6 mice. **** P≤0.0001. Data was analyzed with Kolmogorov–Smirnov test. Scale bars in A/B = 100µm.

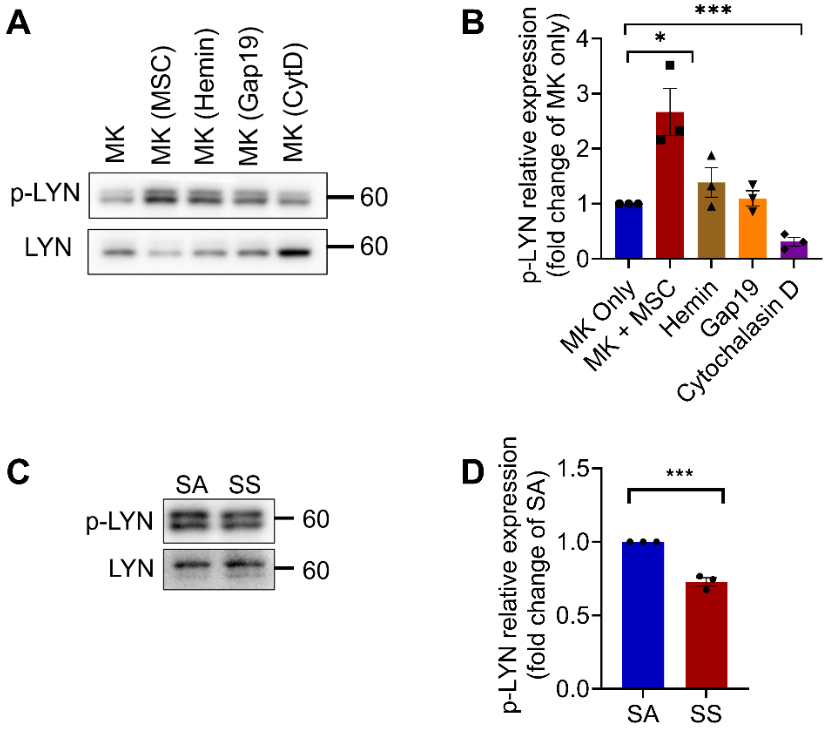

Supplemental Figure 5. p-LYN protein expression in co-cultured MKs and primary murine SCD platelets. Human cord blood derived MKs were cultured together with healthy donor MSCs, Gap19 treated MSCs, cytochalasin D treated MSCs, or cultured alone. **A)** western blot analysis of p-LYN and LYN in cultured MKs. **B)** Quantification of p-LYN protein expression relative to LYN and displayed as fold change of MK only. Townes mice SCD platelets and SA control platelets were analyzed by **C)** western blot for p-LYN and LYN and **D)** quantification of p-LYN protein expression relative to LYN and displayed as fold change of SA. N=3 mice. * P≤0.05 and *** P≤0.001. Data in B was analyzed by 1-way ANOVA with Tukey multiple comparison test and data in D was analyzed with 2-tailed, unpaired Student's *t*-test. Data is presented as mean ± SEM.

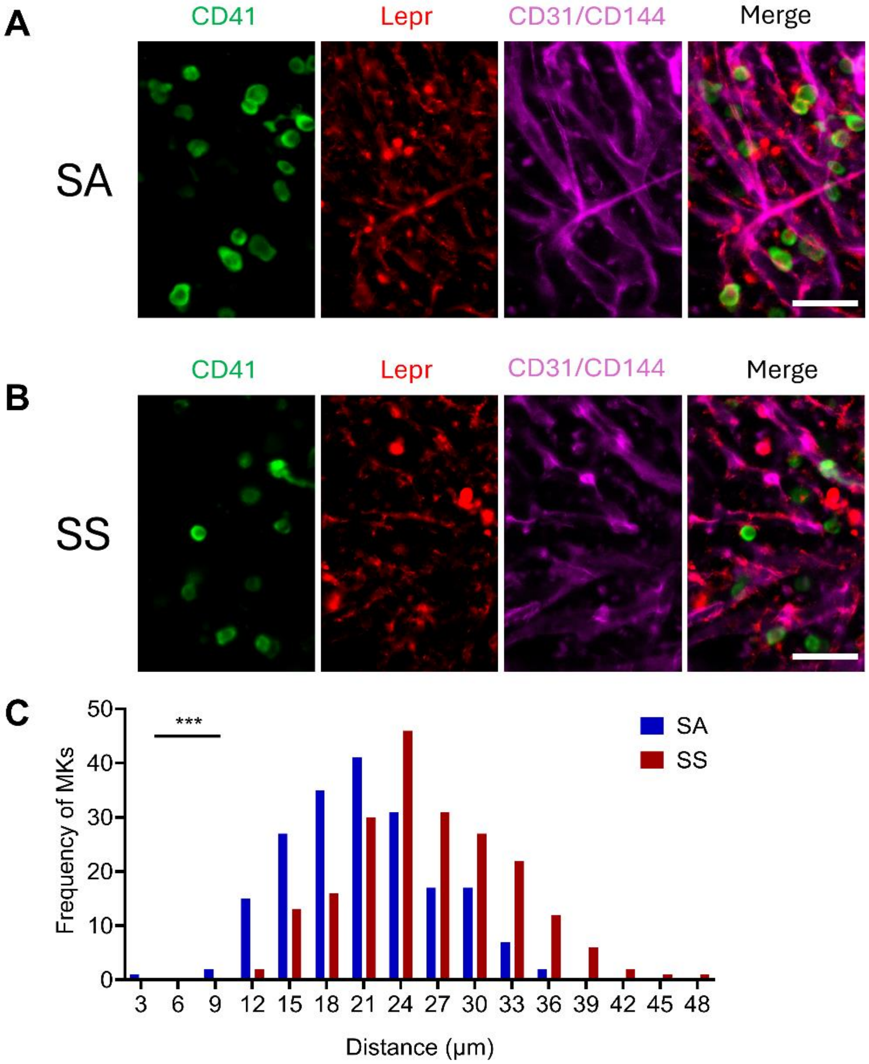

Supplemental Figure 6. Murine SCD MKs localize father away from SCD MSCs compared to SA mice. Whole mount sternum sections stained for CD41, CD31/CD144, and Leptin receptor were imaged by confocal microscopy in **A)** SA mice or **B)** SS Townes mice. **C)** Distribution of MKs in relation to sinusoidal Lepr+ MSCs from SA and SS mice. N = 211 from 6 mice. **** P≤0.0001. Data was analyzed with Kolmogorov–Smirnov test. Scale bars in A/B = 100µm.

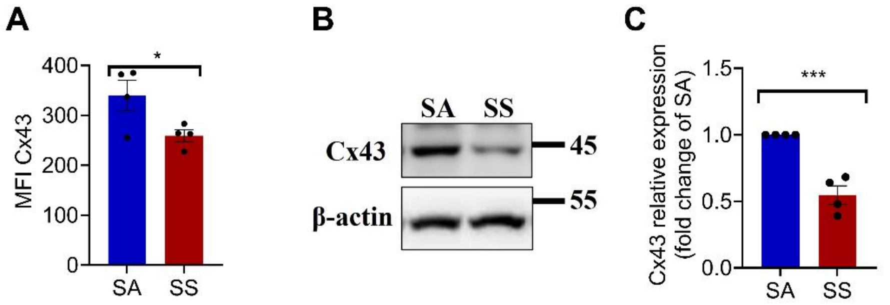

Supplemental Figure 7. CX43 protein expression in SCD mesenchymal stem cells. Townes mice SCD MSCs and SA control MSCs were analyzed by **A)** flow cytometry for CX43 protein expression by mean fluorescence intensity (MFI). **B)** Western blot of CX43 in SCD or SA control bone marrow stromal cells and **C)** quantification of CX43 protein expression relative to β-actin and displayed as fold change of SA. N=4 mice. * P≤0.05 and *** P≤0.001. Data was analyzed with 2-tailed, unpaired Student's *t*-test. Data is presented as mean ± SEM.

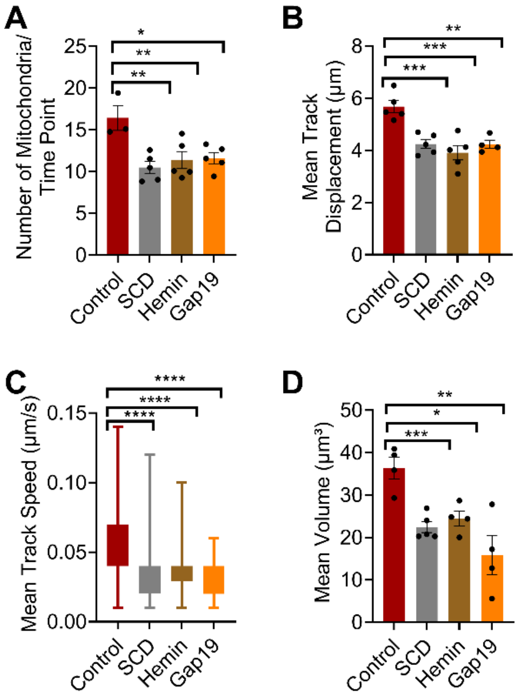

Supplemental Figure 8. Mitochondrial parameters in human MK-MSC co-cultures. Human cord blood derived MKs labeled with Mito Live Orange were co-cultured with unlabeled healthy donor MSCs, SCD MSCs, MSCs treated with 20μM hemin, or 100μM Gap19. Mitochondria acquired by MSCs in time-lapse images were analyzed with Imaris for **A)** number of mitochondria per time point, **B)** mean track displacement, **C)** mean track speed and **D)** mean volume. N=4-5 fields of view. * P≤0.05 ** P≤0.01 *** P≤0.001 **** P≤0.0001. Data were analyzed by 1-way ANOVA with Tukey multiple comparison test. Data is presented as mean ± SEM.

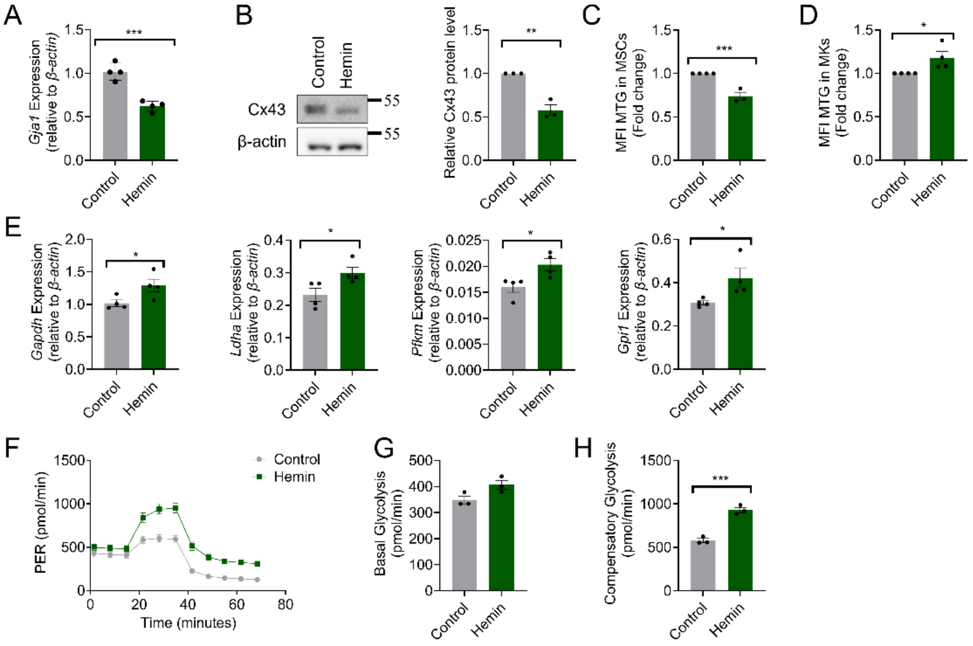

Supplemental Figure 9. Hemin treatment in mice leads to reduced MSC mitochondrial uptake and metabolic changes in MKs and platelets. C57BL/6 mice were treated with 50μmol/kg hemin 3X per week or PBS for 2 weeks. **A)** MSC expression of *Gja1* (Cx43) by RT-PCR. **B)** Western blot of CX43 or β-actin in stromal cells from hemin-treated and control mice. **C)** Flow cytometry analysis of mitotracker green (MTG) mean fluorescence intensity (MFI) in MSCs. **D)** Flow cytometry analysis of MTG MFI signal in MKs. **E)** Sorted MKs from hemin-treated mice or control mice were analyzed by RT-PCR for glycolysis associated gene expression. **F)** Representative seahorse glycolytic rate assay conducted on platelets from hemin treated mice compared to control mice. **G)** Basal glycolysis levels. **H)** Compensatory glycolysis levels. N=3-4 mice. * P≤0.05 ** P≤0.01 *** P≤0.001. Data was analyzed with 2-tailed, unpaired Student's *t*-test. Data is presented as mean ± SEM.
