# Supplementary material for: Megakaryocytes transfer mitochondria to bone marrow mesenchymal stromal cells to lower platelet activation
Source: J Clin Invest. 2025 Feb 27;135(8):e189801. doi: 10.1172/JCI189801 (PMC11996913; doi:10.1172/JCI189801)
Supplement: Unedited blot and gel images [file jci-135-189801-s022.pdf]

Full unedited blot for Supplemental Figure 5A

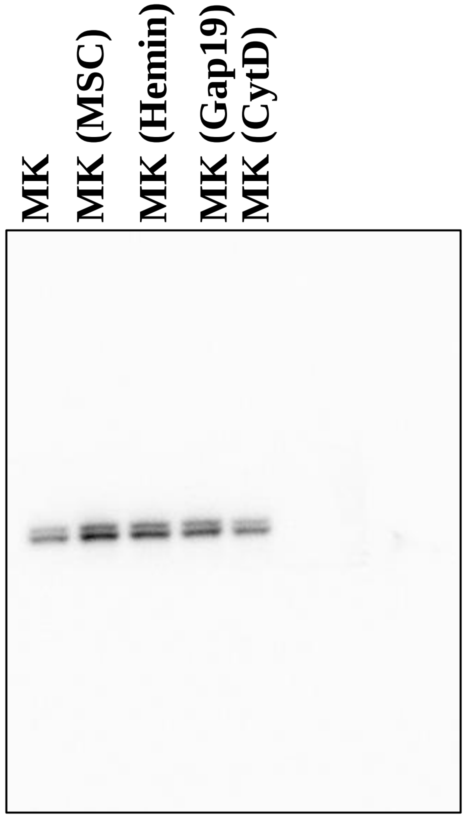

pLyn

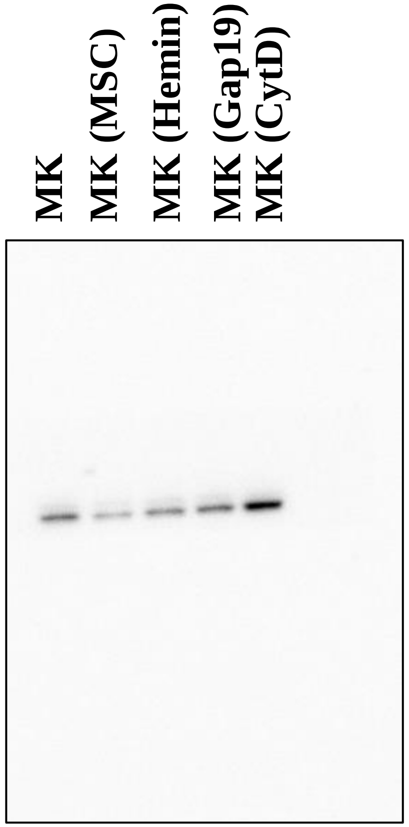

Lyn

Full unedited blot for Supplemental Figure 5C

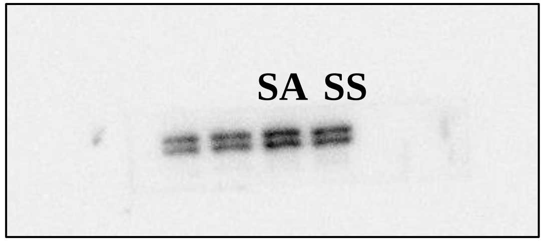

pLyn

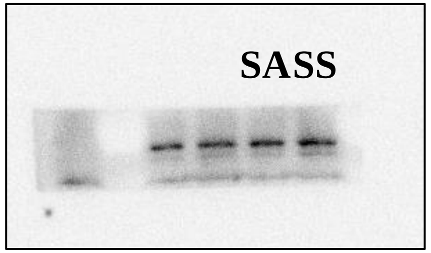

Lyn

Full unedited blot for Supplemental Figure 7B

SA SS

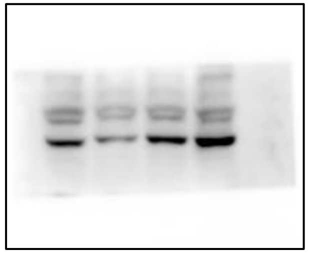

Cx43

SA SS

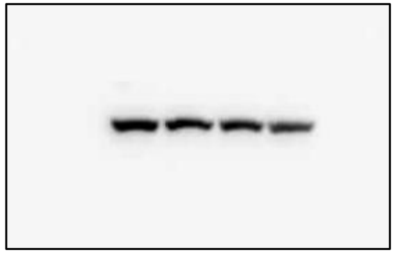

β-actin

Full unedited blot for Supplemental Figure 9B

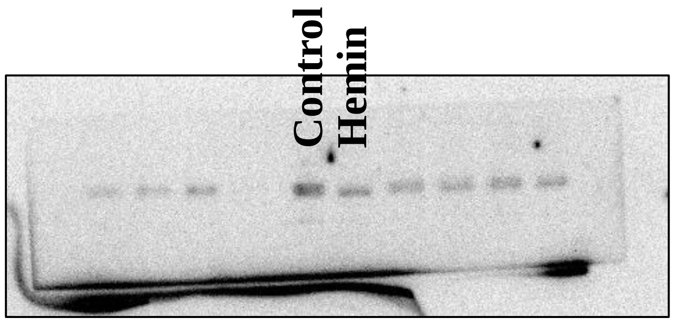

Cx43

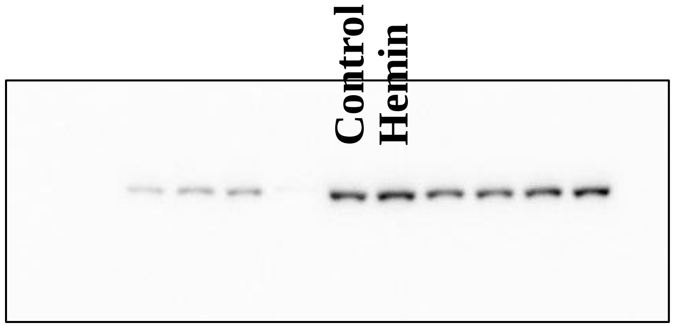

β-actin
